# Mild COVID-19 infection associated with post-COVID-19 condition after 3 months – a questionnaire survey

**DOI:** 10.1080/07853890.2023.2226907

**Published:** 2023-06-20

**Authors:** Stefan Rach, Lisa Kühne, Hajo Zeeb, Wolfgang Ahrens, Ulrike Haug, Hermann Pohlabeln

**Affiliations:** a Leibniz Institute for Prevention Research and Epidemiology – BIPS, Bremen, Germany; b Faculty of Human and Health Sciences, University of Bremen, Bremen, Germany

**Keywords:** SARS-CoV-2, COVID-19, post-COVID-19 condition, Long COVID, cross-sectional, population-based

## Abstract

**Introduction:**

The coronavirus disease 2019 (COVID-19), caused by infection with SARS-CoV-2, can lead to post-COVID-19 condition, a secondary syndrome of persistent and new post-acute symptoms, but evidence on this syndrome is still scarce.

**Methods:**

In a questionnaire survey, residents of the city of Bremen (Germany) with verified SARS-CoV-2 infection were invited to answer questions (online questionnaire or interview) concerning symptoms experienced at the time of infection and at the time of questionnaire completion at least three months later. Main outcome of the analysis was the presence of a post-COVID-19 condition at the time of the interview, defined as the presence of at least two of three leading symptoms: fatigue, breathing difficulties, or cognitive problems.

**Results:**

A post-COVID-19 condition was more likely to be reported if respondents had, at the time of infection, suffered from fatigue (OR 1.75; 95% CI: 1.00, 3.06), breathing difficulties (OR 4.02; 95% CI: 2.80, 5.77), cognitive symptoms (OR 2.98; 95% CI: 1.48, 6.02), or head- & bone aches (OR 2.06; 95% CI: 1.25, 3.42). The odds of developing a post-COVID-19 condition increased with the number of symptoms at infection. Females were more likely to report a post-COVID-19 condition (OR 1.54; 95% CI: 1.05, 2.24). Analyzing only non-hospitalized respondents changed results only slightly.

**Conclusion:**

Our study adds to growing evidence that even a mild course of COVID-19 poses a risk for developing a post-COVID-19 condition. Females and those with initial symptoms including fatigue, breathing difficulties, and cognitive symptoms seem more likely to also experience post COVID-19 symptoms several months after infection.

**KEY MESSAGES**
- Even a mild course of COVID-19 poses a risk for developing a post-COVID-19 condition.
- Females seem more likely to develop a post-COVID-19 condition.
- Those with initial symptoms including fatigue, breathing difficulties, and cognitive symptoms seem more likely to develop a post-COVID-19 condition.

## Introduction

In a considerable proportion of patients with the coronavirus disease 2019 (COVID-19), caused by infection with the severe acute respiratory syndrome coronavirus 2 (SARS-CoV-2), a secondary syndrome of persistent and new post-acute symptoms can be observed that has been coined LongCovid or post-COVID-19 condition [1–4]. Symptoms commonly reported include, but are not limited to, fatigue, shortness of breath, cognitive dysfunction, headache and persistent impairment of taste or smell [5–7]. Typically, patients report that their quality of life decreased considerably [8]. Although a post-COVID-19 condition is more commonly observed in patients severely affected by the initial infection, it is also reported after infections with mild or even no symptoms at all [e.g. 9,10,11]. Given the large number of SARS-CoV-2 infections in the community in Europe and globally, such long-term health consequences may not only impair the quality of life of affected individuals but may also result in high economic costs, e.g. due to sick leaves and long-term treatments, which put substantial strains on the health system.

Here we report results of a questionnaire survey conducted with persons who tested positive for SARS-CoV-2 or were diagnosed with COVID-19. We aimed to investigate which specific symptoms at the time of infection were associated with the reporting of symptoms indicating a post-COVID-19 condition.

## Methods

Persons were eligible for the study if they had been registered by the Public Health Department of the City Municipality of Bremen (Germany) either for having been tested positive for SARS-CoV-2 or for having been diagnosed with COVID-19 by a physician (in the following referred to as SARS-CoV-2 positive). For the sake of readability, the date of registration/date of diagnoses will be referred to as ‘infection’ in the following. Only persons with a date of infection between March, 1^st^, 2020 and January, 31^st^, 2021 who were not registered as being deceased at the time of invitation were invited. The City Municipality of Bremen is the second largest city in Northern Germany (570,000 inhabitants).

Invitations were sent out by the Public Health Department Bremen by landmail between January, 10^th^ and March, 31^st^ 2021. Letters included a cover page with a statement in support of the study from the Public Health Department, an invitation letter, a leaflet with study information from the scientific study team at the Leibniz Institute for Prevention Research and Epidemiology (BIPS), and a consent form together with a stamped return envelope addressed to BIPS. Furthermore, it contained a multi-lingual flyer offering the option to complete the interview in one of seven languages for in addition to German (English, French, Turkish, Bulgarian, Arabic, Polish, Russian). Reminder letters with identical contents were sent out 12 to 16 weeks after the initial invitation letter (median interval 105 days). Persons who returned the completed and signed consent form were contacted by field staff from BIPS at least 90 days after the date of infection (as specified by respondents in the consent form) and were offered the opportunity to either complete an online questionnaire (CAWI) or to participate in a telephone interview (CATI). Contact to conduct the interview was attempted by either landmail or email depending on the preference stated on the consent form and was followed by up to two reminders separated by waiting periods of 14 days (landmail) or 10 days (email). One additional contact attempt by phone was undertaken for persons who provided their phone number on the consent form.

The questionnaire (see Appendix S1 for the German and Appendix S2 for the English version in the supplements) covered personal information, circumstances of the infection, symptoms at the time of infection, symptoms at the time of the interview (called long-term symptoms in the following), information about pre-existing conditions (see Table S1 in the supplements for a complete list), smoking history, education history, current employment, and living conditions. Symptoms were queried in form of a multiple-choice list of 13 symptoms suspected as being typical for COVID-19 at the time of study creation. Additional symptoms could be entered as free text. The corresponding questions explicitly asked to report symptoms ‘at the time of the Corona test/diagnosis and in the first weeks thereafter’ and symptoms at the time of the interview ‘related to the Corona infection’ to avoid that respondents report symptoms unrelated to the COVID-19 infection, for instance, caused by pre-existing conditions. The free texts were first subjected to a word frequency analysis, resulting in the definition of 15 additional symptoms. Afterwards, free texts were screened and coded independently by two reviewers (LK and SR). Disagreements were resolved by discussion until reaching consensus. To reduce the number of variables for analysis, symptoms were grouped into 11 symptom categories (see Table S2 for a complete list of symptom categories). The outcome of interest, post-COVID-19 condition, was defined based on WHO’s clinical case definition [1] as the presence of at least two of three leading symptoms: fatigue, breathing difficulties (shortness of breath), or cognitive problems at the time of the interview. Note that, although WHO’s clinical case definition explicitly requires no minimum number of symptoms, the presence of at least two symptoms was introduced to increase the specificity of the outcome definition. The intended delay of 90 days between infection and interview matches the 3 month criterion cited in WHO’s clinical case definition.

Multivariable logistic regression analysis was applied to assess the association between long-term symptoms and specific symptoms at infection (11 categories). Additional variables included were sex (male vs. female), age (years, continuous), education (International Standard Classification of Education [ISCED]; low: 1, 2; medium: 3, 4; high: 5, 6), smoking status (current/former vs. never), obesity (cutoff BMI >30 kg/m^2^), physician consulted because COVID-19 (yes vs. no), hospitalized for COVID-19 (yes vs. no), 11 pre-existing conditions (yes vs. no; see Table S1 for complete list), and duration between date of infection and date of completing the questionnaire/interview (90–120 days vs. 120–180 days vs. >180 days).

Statistical analyses were performed using the software package SAS, release 9.4 (SAS Institute, Cary, North Carolina, USA) and R (version 4.1.2).

## Results

**Figure 1.**
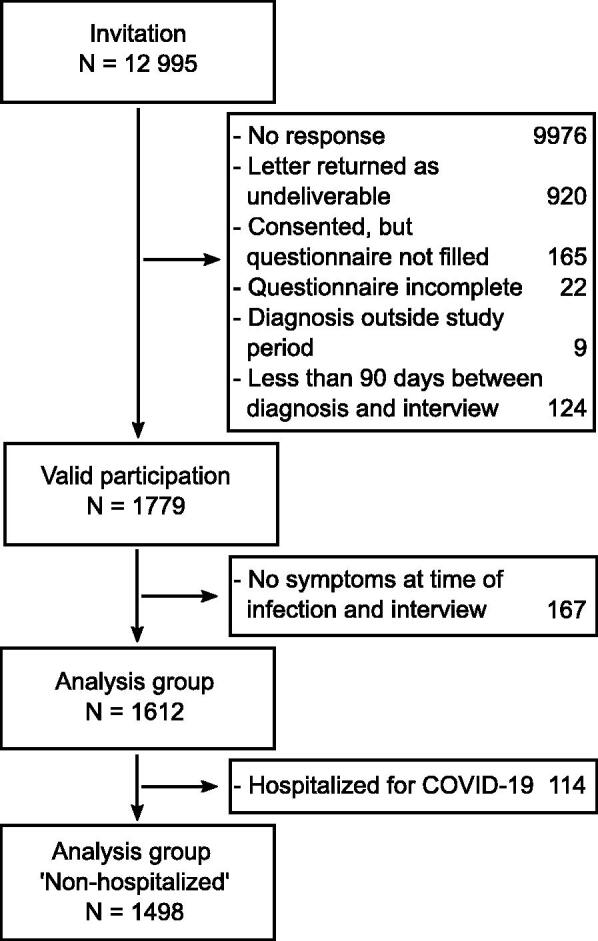
Consort chart.

Out of 12,995 persons invited to the study, 2,009 returned a completed and signed consent form, of which 1,934 eventually completed the questionnaire or the interview, yielding a response proportion of 14.9% (Figure 1). The median interval between infection and interview was 120 days. Persons with an interval between infection and interview shorter than 90 days (*N* = 124) were excluded from further analyses, as were persons with incomplete questionnaires (*N* = 22), and ineligible dates of infection (prior to March, 1st, 2020 or after March, 31st, 2021, *N* = 9), reducing the sample to *N* = 1,779 (13.7% of all persons invited). Overall, 43% of respondents were male (*N* = 760; see Table 1). Education was classified as low in 12% (*N* = 218), as medium in 49% (*N* = 873), as high in 35% (*N* = 630) and missing in 58 respondents.

**Table 1.** Descriptive analysis of the main variables used in the study.

	Analysis group				Study sample			
	Female		Male		Female		Male	
	*n*	%	*n*	%	*n*	%	*n*	%
Age								
	55	5.8	41	6.1	72	7.1	55	7.2
18–29	138	14.6	85	12.7	146	14.3	91	12.0
30–39	129	13.7	107	16.0	136	13.3	114	15.0
40–49	159	16.9	94	14.1	167	16.4	106	13.9
50–59	228	24.2	164	24.5	239	23.5	176	23.2
60–69	140	14.8	89	13.3	151	14.8	108	14.2
70–79	54	5.7	61	9.1	59	5.8	76	10.0
80+	40	4.2	28	4.2	49	4.8	34	4.5
Education (ISCED)								
Low (1, 2)	113	12.0	71	10.6	134	13.2	84	11.1
Medium (3, 4)	495	52.5	307	45.9	528	51.8	345	45.4
High (5, 6)	312	33.1	269	40.2	329	32.3	301	39.6
Missing	23	2.4	22	3.3	28	2.7	30	3.9
Weight status (BMI, kg/m²)^a^								
Normal (<25)	512	54.3	281	42.0	564	55.3	318	41.8
Overweight (25–30)	243	25.8	265	39.6	255	25.0	298	39.2
Obese (30+)	186	19.7	120	17.9	197	19.3	140	18.4
Missing	2	0.2	3	0.4	3	0.3	4	0.5
Smoking status								
Never smoked	563	59.7	373	55.8	619	60.7	420	55.3
Current or former	380	40.3	296	44.2	400	39.3	340	44.7
Pre-existing conditions								
0	503	53.3	340	50.8	549	53.9	381	50.1
1	217	23.0	164	24.5	235	23.1	185	24.3
2	118	12.5	85	12.7	121	11.9	99	13.0
	105	11.1	80	12.0	114	11.2	95	12.5
Physician consulted because of Covid-19								
No	698	74.0	543	81.2	774	76.0	634	83.4
Yes	245	26.0	126	18.8	245	24.0	126	16.6
Hospitalized for Covid-19								
No	880	93.3	618	92.4	956	93.8	709	93.3
Yes	63	6.7	51	7.6	63	6.2	51	6.7
Number of symptoms at time of infection								
0	0	0.0	0	0.0	76	7.5	91	12.0
1–2	191	20.3	178	26.6	191	18.7	178	23.4
3–4	339	35.9	266	39.8	339	33.3	266	35.0
5–6	309	32.8	192	28.7	309	30.3	192	25.3
7+	104	11.0	33	4.9	104	10.2	33	4.3
Number of symptoms at time of interview								
0	453	48.0	418	62.5	529	51.9	509	67.0
1–2	328	34.8	191	28.6	328	32.2	191	25.1
3–4	133	14.1	54	8.1	133	13.1	54	7.1
5+	29	3.1	6	0.9	28	2.8	6	0.8
All	943	100.0	669	100.0	1019	100.0	760	100.0

^a^Calculated from weight and height as reported in questionnaire.

Due to data protection regulations, the Public Health Department Bremen provided only the number of invitations sent, but no information on the age or sex distribution of invited persons. Therefore, characteristics of the study sample could only be compared to official data on confirmed cases of SARS-CoV-2 in the City of Bremen as provided by German federal authorities (Robert Koch Institute, Berlin) [12]. Compared to all confirmed cases of SARS-CoV-2 officially registered in the City Municipality of Bremen between March, 1st, 2020 and January, 31st, 2021, females were slightly overrepresented in our sample (57% vs. 51%) as were persons in age groups 35–59 and 60–79. Persons in age group 15–34 were slightly underrepresented (Table 2).

**Table 2.** Sample composition stratified by age and sex in comparison to all confirmed cases of SARSCoV-2 officially registered by RKI [12] in the City of Bremen (Germany) between March, 1^st^, 2020 and January, 31^st^, 2021.

Age group	Female			Male			Total
	*N*	%	% RKI	*N*	%	% RKI	
0 - 4	4	0.00	0.01	7	0.00	0.01	11
5 - 14	47	0.03	0.03	33	0.02	0.03	80
15 - 34	239	0.13	0.18	177	0.10	0.19	416
35 - 59	470	0.26	0.19	325	0.18	0.19	795
60 - 79	210	0.12	0.06	184	0.10	0.06	394
80+	49	0.03	0.04	34	0.02	0.02	83
Sum	1019	0.57	0.51	760	0.43	0.49	1779

**Figure 2.**
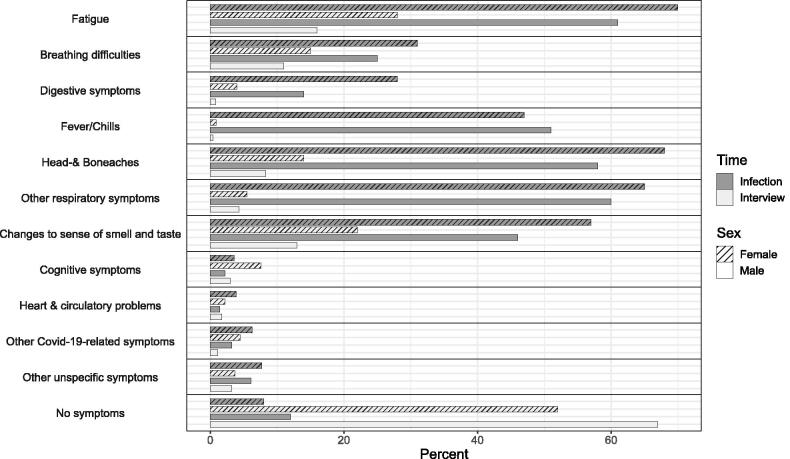
Symptoms reported at the time of infection (dark gray) and the time of interview (light gray) for females (hatching) and males (no hatching).

Almost all respondents reported at least one symptom at the time of infection (*N* = 1,612; 90.6%). Symptoms reported most frequently (Table S2) were fatigue (*N* = 1,177; 66.2%), head & bone aches (*N* = 1,131; 63.6%), other respiratory problems (sore throat, cough, runny nose; *N* = 1,116; 62.7%), changes to sense of smell and taste (*N* = 928; 52.2%), and fever/chills (*N* = 870; 48.9%) without marked differences between males and females (Figure 2). Consultations with physicians (Table 1) were more frequently reported by females (*N* = 245; 24.0%) than by males (*N* = 126; 16.6%), whereas no marked differences were found in the reporting of hospitalizations between females (*N* = 63; 6.2%) and males (*N* = 51; 6.7%).

741 respondents (41.7%) reported to still have symptoms at the time of the interview. Most frequently reported symptoms (Table S2) were fatigue (*N* = 411; 23.1%), changes to sense of smell and taste (*N* = 325; 18.3%), breathing difficulties (*N* = 237; 13.3%), and head & bone aches (*N* = 204; 11.5%), with females reporting symptoms more often (Figure 2). None of the 167 respondents without symptoms at the time of infection reported any symptoms at the time of the interview.

**Figure 3.**
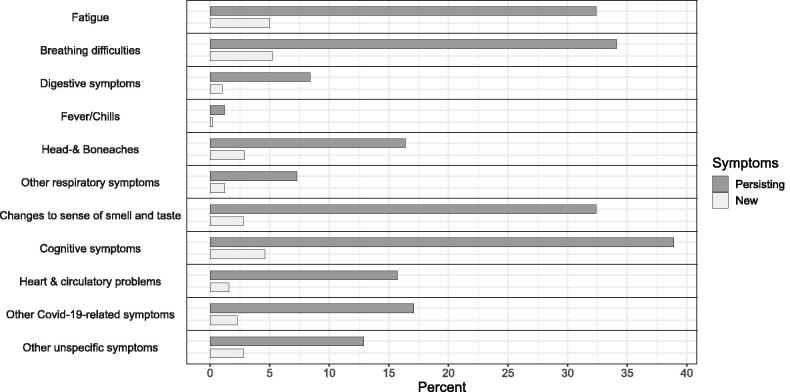
Percentage of symptoms reported at time of infection that were still persistent at time of the interview (dark gray) and percentage of symptoms that were newly reported at the time of interview (light gray).

Persistence of symptoms (Figure 3), that is, the percentage of symptoms reported at the time of infection that were still persisting at the time of the interview, was highest for cognitive symptoms (38.9%), breathing difficulties (34.1%), fatigue (32.4%), and change of sense of taste and smell (32.4%).

The frequency of symptoms that were reported at the time of interview, but not initially at infection was highest for breathing difficulties (5.2%), fatigue (5.0%), and cognitive symptoms (4.6%).

To investigate the association between a post-COVID-19 condition (2 out of 3 of fatigue, shortness of breath, and cognitive difficulty at the time of the interview) and presence of symptoms at infection, the analysis was limited to those with symptoms at least one time point. Respondents that neither reported symptoms at infection, nor at the time of interview (*N* = 167; 45.5% females) were excluded from further analysis, reducing the sample to 1,612 respondents for the main analysis. In this sample, 203 respondents (12.6%) met our definition of the post-COVID condition, of which 145 (71.4%) were female (Table 3). The post-COVID-19 condition was more likely to be reported if respondents had, at the time of infection, suffered from fatigue (OR 1.75; 95% CI: 1.00, 3.06), breathing difficulties (OR 4.02; 95% CI: 2.80, 5.77), cognitive symptoms (OR 2.98; 95% CI: 1.48, 6.02), or head- & bone aches (OR 2.06; 95% CI: 1.25, 3.42). Although changes to sense of smell and taste showed a high persistence from infection to interview (32.4%), the respective odds for a post-COVID-19 condition were not elevated (OR: 0.91; 95% CI: 0.64, 1.31). Furthermore, none of the preexisting conditions queried in the survey were associated with the post COVID-19 condition. Females were more likely to report long-term symptoms consistent with a post-COVID-19 condition (OR 1.54; 95% CI: 1.05, 2.24), as were respondents who had consulted a physician (OR 2.25; 95% CI: 1.54, 3.30) or who were hospitalized (OR 3.68; 95% CI: 2.08, 6.49). In respondents where the duration between infection and interview exceeded 180 days, long-term symptoms were less frequently reported (OR 0.63; 95% CI: 0.42, 0.95) as compared to respondents completing the questionnaire within 90–120 days (Table 3).

**Table 3.** Odds ratios (95% CIs) for reporting a post-COVID-19 for the analysis group (*N* = 1,612) condition estimated from a multivariable logistic regression model.

	Post-COVID-19 condition					
	No		Yes			
	n	%	n	%	OR	95% CI
**Sex**						
Male	611	91.3	58	8.7	Ref.	
Female	798	84.6	145	15.4	1.54	(1.05, 2.24)
**Age (mean, sd)**	46.6	18.9	51.7	15.1	1.00	(0.99, 1.01)
**Fatigue**						
No	417	95.9	18	4.1	Ref.	
Yes	992	84.3	185	15.7	1.75	(1.00, 3.06)
**Breathing difficulties**						
No	1051	94.3	63	5.7	Ref.	
Yes	358	71.9	140	28.1	4.02	(2.80, 5.77)
**Cognitive symptoms**						
No	1377	88.4	181	11.6	Ref.	
Yes	32	59.3	22	40.7	2.98	(1.48, 6.02)
**Digestive symptoms**						
No	1098	90.2	119	9.8	Ref.	
Yes	311	78.7	84	21.3	1.24	(0.86, 1.80)
**Fever/Chills**						
No	665	89.6	77	10.4	Ref.	
Yes	744	85.5	126	14.5	0.87	(0.61, 1.25)
**Head-& Boneaches**						
No	454	94.4	27	5.6	Ref.	
Yes	955	84.4	176	15.6	2.06	(1.25, 3.42)
**Other respiratory symptoms**						
No	451	90.9	45	9.1	Ref.	
Yes	958	85.8	158	14.2	1.16	(0.76, 1.76)
**Changes to sense of smell and taste **						
No	609	89.0	75	11.0	Ref.	
Yes	800	86.2	128	13.8	0.91	(0.64, 1.31)
**Heart & circulatory problems**						
No	1375	88.1	186	11.9	Ref.	
Yes	34	66.7	17	33.3	1.51	(0.71, 3.24)
**Other Covid-19-related symptoms**						
No	1340	87.9	184	12.1	Ref.	
Yes	69	78.4	19	21.6	0.84	(0.44, 1.61)
**Other unspecific symptoms**						
No	1314	88.3	174	11.7	Ref.	
Yes	95	76.6	29	23.4	1.37	(0.78, 2.39)
**High blood pressure or hypertension**						
No	1041	88.6	134	11.4	Ref.	
Yes	368	84.2	69	15.8	0.87	(0.58, 1.31)
**Other cardiovascular diseases** ^a^						
No	1307	88.0	179	12.0	Ref.	
Yes	102	81.0	24	19.0	0.80	(0.43, 1.50)
**Diabetes mellitus**						
No	1335	87.7	187	12.3	Ref.	
Yes	74	82.2	16	17.8	0.72	(0.36, 1.44)
**Asthma**						
No	1268	88.3	168	11.7	Ref.	
Yes	141	80.1	35	19.9	1.17	(0.67, 2.05)
**Chronic lung disease (e.g. COPD)**						
No	1302	88.6	168	11.4	Ref.	
Yes	107	75.4	35	24.6	1.49	(0.83, 2.66)
**Chronic bronchitis**						
No	1345	88.4	177	11.6	Ref.	
Yes	64	71.1	26	28.9	1.30	(0.71, 2.38)
**Cancer**						
No	1323	88.0	180	12.0	Ref.	
Yes	86	78.9	23	21.1	1.65	(0.79, 3.48)
**Currently receiving medical treatment for cancer**						
No	1377	87.8	192	12.2	Ref.	
Yes	32	74.4	11	25.6	0.83	(0.28, 2.51)
**Weakened immune system** ^b^						
No	1322	88.5	172	11.5	Ref.	
Yes	87	73.7	31	26.3	1.57	(0.92, 2.69)
**Chronic liver disease**						
No	1385	87.6	196	12.4	Ref.	
Yes	24	77.4	7	22.6	0.99	(0.34, 2.90)
**Adiposity (diagnosed by physician)**						
No	1313	88.1	177	11.9	Ref.	
Yes	96	78.7	26	21.3	1.06	(0.56, 2.01)
**Education (ISCED)**						
Low (ISCED 1, 2)	161	87.5	23	12.5	1.39	(0.74, 2.58)
Medium (ISCED 3, 4)	683	85.2	119	14.8	1.24	(0.84, 1.84)
High (ISCED 5, 6)	524	90.2	57	9.8	Ref.	
Missing	41	91.1	4	8.9	1.04	(0.28, 3.90)
**Obese (BMI >30 kg/m²)** ^c^						
No	1164	89.1	142	10.9	Ref.	
Yes	245	80.1	61	19.9	1.53	(0.97, 2.41)
**Smoking status**						
Never smoked	837	89.4	99	10.6	Ref.	
Current or ex-smoker	572	84.6	104	15.4	1.19	(0.84, 1.69)
**Physician consulted**						
No	1121	90.3	120	9.7	Ref.	
Yes	288	77.6	83	22.4	2.25	(1.54, 3.30)
**Hospitalized**						
No	1334	89.1	164	10.9	Ref.	
Yes	75	65.8	39	34.2	3.68	(2.08, 6.49)
**Time interval infection to interview**						
90–120 days	481	85.4	82	14.6	Ref.	
120–180 days	425	87.8	59	12.2	0.71	(0.47, 1.08)
	503	89.0	62	11.0	0.63	(0.42, 0.95)
**All**	1409	87.4	203	12.6		

^a^Includes circulatory problems of the heart, stenosis of the coronary arteries, angina pectoris, heart attack, heart failure or heart insufficiency, stroke.

^b^Medical conditions associated with weakened immune system or under medications that weaken the immune system.

^c^Calculated from weight and height as reported in questionnaire.

Repeating the same analysis with symptom counts instead of individual symptoms (see Table S3) revealed that, compared to the reference group with 1–2 symptoms, the odds of a post-COVID-19 condition gradually increased with the number of symptoms at infection. For persons with 3–4 symptoms the OR was 1.80 (95% CI: 0.92, 3.5), for persons with 5–6 symptoms the OR was 5.59 (95% CI: 2.97, 10.53), and for those with more than 7 symptoms the OR was 7.52 (95% CI: 3.68, 15.39).

Results only changed slightly when the analysis sample was restricted to respondents who were not hospitalized (*N* = 1,498; see Table S4). In the non-hospitalized sample, 164 respondents (10.9%) met the conditions of a post-COVID condition, of which 122 were female (74.4%). Elevated ORs for the post-COVID-19 condition were still observed for respondents reporting breathing difficulties (OR 4.27; 95% CI: 2.88, 6.32), cognitive symptoms (OR 3.13; 95% CI: 1.38, 7.12), or head- & bone aches (OR 2.28; 95% CI: 1.26, 4.14), as well as for females (OR 1.58; 95% CI: 1.03, 2.40) and respondents who consulted a physician (OR 2.22; 95% CI: 1.50, 3.29). In addition, respondents with a history of cancer were more likely to report a post-COVID-19 condition (OR 2.48; 95% CI: 1.11, 5.52) in non-hospitalized respondents.

## Discussion

In this questionnaire survey 1,779 persons with confirmed infection with SARS-CoV-2 reported symptoms they experienced at the time of infection and at the time of an interview at least three months later. The focus of our analysis was the investigation of associations between symptoms present at the time of infection with SARS-CoV-2 and the likely presence of a post-COVID-19 condition, defined here as the presence of at least two of the three leading symptoms fatigue, breathing difficulties, and cognitive problems at the time of the interview. Persons reporting breathing difficulties at the time of infection had a four times higher odds of developing post-COVID-19 condition as compared to persons without this symptom. For cognitive symptoms, fatigue or head- & bone aches the odds were two to three times higher as compared to those without these symptoms. Females were more likely to report long-term symptoms consistent with a post-COVID-19 condition. The odds of developing post-COVID-19 substantially increased with the number of symptoms reported at infection. No respondent without symptoms at the time of infection reported any symptoms at the time of infection.

The proportion of respondents classified as suffering from a post-COVID-19 condition, as well as the frequency of main symptoms reported in this survey confirms previous studies [e.g. 5,9,10,13–15]. Only cognitive symptoms were reported less frequently in our sample as compared to previous studies. This difference might be explained by the fact that cognitive symptoms were not among the multiple-choice options listed in our questionnaire, but instead derived from free texts entered by respondents.

Although having consulted a physician or being hospitalized increased the odds of belonging to the post-COVID-19 condition group, the observed associations did not differ markedly when only non-hospitalized respondents were included in the analysis, suggesting that already mild courses of the disease might have long lasting consequences. This observation is supported by earlier reports in which the frequency of patients with mild COVID-19 who developed persistent symptoms ranged from 10% to 35% [cf. 11 for a review]. Other reports indicate that, even one year after infection, non-hospitalized COVID-19 patients rated their health worse than before their infection more often than controls or the general population [10].

The observation that the odds of a post-COVID-19 condition was lower for respondents where the duration between infection and interview exceeded 180 days is consistent with findings reported for hospitalized COVID-19 patients [16,17].

In our study, females were more likely to have post-COVID-19 condition, adding to the growing body of evidence that women are more likely to be affected by this condition [2,9,15]. This observation might be explained by a sex specific manifestation of symptoms, sex differences in symptom reporting and gender-related social factors as well as sex differences in the subjective perception of symptoms and pain [18].

Overall, the study adds to evidence indicating that long-term health consequences, particularly fatigue, are frequent even among persons with a mild course of COVID-19. To which degree the severity of these long-term consequences lead to increased utilization of medical services will determine the resulting burden for health systems. While there are many post-hospitalization or electronic health record-based studies on post-Covid-19 conditions, the fact that we used a population-based approach is a strength of our study as it allows to investigate effects of COVID-19 to which the health system might be agnostic. In our study only 122 of the 203 respondents in the post-COVID-19 condition group sought medical care at the time of infection (83 consulted a physician, 39 were hospitalized; cf. Table 3). This means that a large fraction of persons suffering from post COVID-19 condition may escape documentation of their initial infection in medical health records. This further complicates the identification of post COVID-19 condition after mild infection in health record data and, in particular, its differentiation from similar conditions [19–23].

Known limitations of this study include that our sample did not include a control group of individuals without history of SARS-CoV-2 infection. Furthermore, information on both time points of interest (infection, interview) were obtained at the same point in time, that is, respondents had to recall symptoms at the time of infection, with a potential for misclassification and recall bias. Also the severity of symptoms or impact on functioning was not assessed. The fact that not all symptoms were included in the questionnaire (e.g. cognitive symptoms) and had to be coded from free texts, may have resulted in underreporting. The observation that they were reported in relevant numbers albeit not being specifically queried, however, sheds light on their relevance in the context of post-COVID-19 conditions. Information about SARS-CoV-2 infection itself can be considered as valid as it was officially verified by health authorities for each individual case. Data refer to a time when the wild-type SARS-CoV-2 was predominant in the study region and vaccinations were not yet available to the general public. Given the fact, however, that in February 2023 about 30% of the world’s population has not been vaccinated yet, including almost 75% of people in low-income countries [24], research in unvaccinated populations might not be without merit for some time to come. Finally, with a response proportion of only 15%, selection effects may have biased the study sample, although associations between acute and persistent symptoms are less likely to be biased due to selection, as compared to prevalence estimates (which could not be reported in this study because we lacked age/sex specific response proportions for reweighting).

In conclusion, our study adds to the growing body of evidence that even a mild course of COVID-19 poses a risk for developing post-COVID 19 symptoms (fatigue, breathing difficulties, and cognitive symptoms). Females and those with symptoms including fatigue, breathing difficulties and cognitive symptoms at the time of infection seem more likely to experience post COVID-19 symptoms several months after infection.

## Supplementary Material

Supplemental Material

## Data Availability

Datasets used in this study are available from the corresponding author (SR) upon reasonable request.
