# Supplementary material for: Mild COVID-19 infection associated with post-COVID-19 condition after 3 months – a questionnaire survey
Source: Ann Med. 2023 Jun 20;55(1):2226907. doi: 10.1080/07853890.2023.2226907 (PMC10283437; doi:10.1080/07853890.2023.2226907)
Supplement: Supplemental Material [file IANN_A_2226907_SM0857.pdf]

Supplementary Information

Mild COVID-19 infection associated with Post-COVID-19 condition after 3 months – a questionnaire survey

Stefan Rach[1,+], Lisa Kühne[1], Hajo Zeeb[1,2], Wolfgang Ahrens[1], Ulrike Haug[1,2,#], Hermann Pohlabeln[1,#]

[1] Leibniz Institute for Prevention Research and Epidemiology – BIPS, Bremen, Germany

[2] Faculty of Human and Health Sciences, University of Bremen, Bremen, Germany.

[+]Corresponding authors:
Stefan Rach (rach@leibniz-bips.de, sec-epi@leibniz-bips.de)
Leibniz Institute for Prevention Research and Epidemiology - BIPS
Achterstr. 30
28359 Bremen, Germany

Contents

Table S1 Self-reported preexisting conditions, stratified by sex

Table S2 Frequency of symptoms in the analysis group gathered as multiple choice questions (MC) and coded from free texts (FT) and corresponding symptom categories. Note that frequencies of categories (bold font) deviate from the sum of sub categories (light font), because multiple symptoms within a category could be reported by respondents

Table S3 Odds ratios (95% CIs) for reporting a post-COVID-19 for the analysis group (N = 1.612) condition estimated from a multivariable logistic regression model with symptom counts instead of individual symptoms at infection

Table S4 Odds ratios (95% CIs) for reporting a Post-COVID-19 condition in non-hospitalized respondents (N = 1,498) estimated from a multivariable logistic regression model

Appendix S1 Questionnaire German (CAPI Version)

Appendix S2 Questionnaire English (CAPI Version)

Table S1 Self-reported preexisting conditions, stratified by sex

	Analysis group				Study sample			
	Female		Male		Female		Male	
	n	%	n	%	n	%	n	%
High blood pressure or hypertension	233	24.7	204	30.5	249	24.4	240	31.6
Other cardiovascular diseases[a]	60	6.4	66	9.9	65	6.4	82	10.8
Circulatory problems of the heart, stenosis of the coronary arteries, angina pectoris	27	2.9	44	6.6	28	2.7	52	6.8
Heart attack	10	1.1	24	3.6	10	1.0	31	4.1
Heart failure or heart insufficiency	26	2.8	32	4.8	30	2.9	39	5.1
Stroke	16	1.7	13	1.9	18	1.8	15	2.0
Diabetes mellitus	47	5.0	43	6.4	52	5.1	53	7.0
Asthma	105	11.1	71	10.6	110	10.8	74	9.7
Chronic lung disease (e.g., COPD)	83	8.8	59	8.8	84	8.2	63	8.3
Chronic bronchitis	68	7.2	22	3.3	70	6.9	25	3.3
Cancer	64	6.8	45	6.7	76	7.5	56	7.4
Currently receiving medical treatment for cancer	25	2.7	18	2.7	28	2.7	22	2.9
Weakened immune system[b]	82	8.7	36	5.4	85	8.3	43	5.7
Chronic liver disease	15	1.6	16	2.4	17	1.7	18	2.4
Adiposity (diagnosed by physician)	85	9.0	37	5.5	92	9.0	41	5.4

[a] To reduce the number of variables for analysis, categories “circulatory problems of the heart”, stenosis of the coronary arteries, angina pectoris”, “heart attack, heart failure or heart insufficiency”, and “stroke” were combined to “Other cardiovascular diseases”. Note that frequencies deviate from the sum of sub categories since multiple mentions were possible.

[b] Medical conditions associated with weakened immune system or under medications that weaken the immune system

Table S2 Frequency of symptoms in the analysis group gathered as multiple choice questions (MC) and coded from free texts (FT) and corresponding symptom categories. Note that frequencies of categories (bold font) deviate from the sum of sub categories (light font), because multiple symptoms within a category could be reported by respondents

Symptoms	Format	At infection				At interview			
		Female		Male		Female		Male	
		n	%	n	%	n	%	n	%
**Fatigue**	**MC**	**715**	**70.2**	**462**	**60.8**	**286**	**28.1**	**125**	**16.4**
**Breathing difficulties**	**MC**	**311**	**30.5**	**187**	**24.6**	**155**	**15.2**	**82**	**10.8**
**Digestive symptoms**		**289**	**28.4**	**106**	**13.9**	**41**	**4.0**	**6**	**0.8**
Diarrhea	MC	174	17.1	70	9.2	13	1.3	2	0.3
Nausea	MC	186	18.3	40	5.3	17	1.7	4	0.5
Abdominal, bowel, bladder or kidney symptoms	FT	30	2.9	13	1.7	17	1.7	2	0.3
**Fever/Chills**		**482**	**47.3**	**388**	**51.1**	**9**	**0.9**	**3**	**0.4**
Fever	MC	409	40.1	344	45.3	2	0.2	0	0.0
Chills	MC	242	23.7	166	21.8	5	0.5	1	0.1
Flushing, sweats	FT	13	1.3	16	2.1	3	0.3	2	0.3
**Head- & Boneaches**		**691**	**67.8**	**440**	**57.9**	**141**	**13.8**	**63**	**8.3**
Boneaches	MC	518	50.8	326	42.9	75	7.4	46	6.1
Headaches	MC	569	55.8	324	42.6	93	9.1	29	3.8
**Other respiratory symptoms**		**658**	**64.6**	**458**	**60.3**	**56**	**5.5**	**33**	**4.3**
Sore throat	MC	383	37.6	252	33.2	20	2.0	17	2.2
Cough	MC	412	40.4	319	42.0	35	3.4	17	2.2
Runny nose	MC	318	31.2	207	27.2	13	1.3	10	1.3
**Changes to sense of smell or taste**		**580**	**56.9**	**348**	**45.8**	**225**	**22.1**	**100**	**13.2**
Change to sense of taste	MC	519	50.9	298	39.2	158	15.5	58	7.6
Change to sense of smell	MC	515	50.5	313	41.2	199	19.5	90	11.8
**Cognitive symptoms**	**FT**	**37**	**3.6**	**17**	**2.2**	**77**	**7.6**	**23**	**3.0**
**Other Covid-19-related symptoms**		**64**	**6.3**	**24**	**3.2**	**46**	**4.5**	**8**	**1.1**
Dizziness	FT	37	3.6	12	1.6	17	1.7	1	0.1
Hair loss	FT	9	0.9	2	0.3	24	2.4	4	0.5
Dermatological skin symptoms	FT	14	1.4	3	0.4	6	0.6	1	0.1
Sensory skin symptoms	FT	10	1.0	8	1.1	4	0.4	3	0.4
**Cardiovascular symptoms**		**40**	**3.9**	**11**	**1.4**	**22**	**2.2**	**13**	**1.7**
Tightness of chest	FT	16	1.6	4	0.5	4	0.4	5	0.7
Heart problems	FT	17	1.7	5	0.7	17	1.7	9	1.2
Circulatory problems	FT	12	1.2	3	0.4	2	0.2	0	0.0
**Other non-specific symptoms**		**78**	**7.7**	**46**	**6.1**	**38**	**3.7**	**24**	**3.2**
Loss of appetite	FT	30	2.9	24	3.2	3	0.3	1	0.1
Mental difficulties	FT	14	1.4	6	0.8	15	1.5	11	1.4
Sleep difficulties	FT	6	0.6	9	1.2	11	1.1	4	0.5
Nose & ear related symptoms	FT	21	2.1	5	0.7	9	0.9	4	0.5
Eye related symptoms	FT	18	1.8	8	1.1	8	0.8	6	0.8
**No symptoms**		**76**	**7.5**	**91**	**12.0**	**529**	**51.9**	**509**	**67.0**
**All**		**1019**	**100**	**760**	**100**	**1019**	**100**	**760**	**100**

Table S3 Odds ratios (95% CIs) for reporting a post-COVID-19 for the analysis group (N = 1.612) condition estimated from a multivariable logistic regression model with symptom counts instead of individual symptoms at infection

	Post-COVID-19 condition					
	No		Yes		OR	95% CI
	n	%	n	%		
**Sex**						
Male	611	91.3	58	8.7	Ref	
Female	798	84.6	145	15.4	1.54	(1.08, 2.21)
**Age (mean, sd)**	46.6	18.9	51.7	15.1	1.00	(0.99, 1.01)
**Symtoms at infection**						
1-2	357	96.7	12	3.3	Ref.	
3-4	561	92.7	44	7.3	1.80	(0.92, 3.51)
5-6	398	79.4	103	20.6	5.59	(2.97, 10.53)
7+	93	67.9	44	32.1	7.52	(3.68, 15.39)
**High blood pressure or hypertension**						
No	1041	88.6	134	11.4	Ref.	
Yes	368	84.2	69	15.8	0.85	(0.57, 1.27)
**Other cardiovascular diseases**[a]						
No	1307	88.0	179	12.0	Ref.	
Yes	102	81.0	24	19.0	0.82	(0.45, 1.49)
**Diabetes mellitus**						
No	1335	87.7	187	12.3	Ref.	
Yes	74	82.2	16	17.8	0.81	(0.42, 1.57)
**Asthma**						
No	1268	88.3	168	11.7	Ref.	
Yes	141	80.1	35	19.9	1.19	(0.69, 2.06)
**Chronic lung disease (e.g., COPD)**						
No	1302	88.6	168	11.4	Ref.	
Yes	107	75.4	35	24.6	1.60	(0.91, 2.81)
**Chronic bronchitis**						
No	1345	88.4	177	11.6	Ref.	
Yes	64	71.1	26	28.9	1.41	(0.79, 2.53)
**Cancer**						
No	1323	88.0	180	12.0	Ref.	
Yes	86	78.9	23	21.1	1.40	(0.68, 2.91)
**Currently receiving medical treatment for cancer**						
No	1377	87.8	192	12.2	Ref.	
Yes	32	74.4	11	25.6	1.09	(0.38, 3.12)
**Weakened immune system**[b]						
No	1322	88.5	172	11.5	Ref.	
Yes	87	73.7	31	26.3	1.54	(0.92, 2.58)
**Chronic liver disease**						
No	1385	87.6	196	12.4	Ref.	
Yes	24	77.4	7	22.6	1.15	(0.41, 3.19)
**Adiposity (diagnosed by physician)**						
No	1313	88.1	177	11.9	Ref.	
Yes	96	78.7	26	21.3	1.00	(0.55, 1.82)
**Education (ISCED)**						
Low (ISCED 1, 2)	161	87.5	23	12.5	1.36	(0.75, 2.47)
Medium (ISCED 3, 4)	683	85.2	119	14.8	1.22	(0.83, 1.78)
High (ISCED 5, 6)	524	90.2	57	9.8	Ref.	
Missing	41	91.1	4	8.9	0.81	(0.22, 2.92)
**Obese (BMI >30 kg/m^2)**[c]						
No	1164	89.1	142	10.9	Ref.	
Yes	245	80.1	61	19.9	1.66	(1.06, 2.58)

**Smoking status**						
Never smoked	837	89.4	99	10.6	Ref.	
Current or ex-smoker	572	84.6	104	15.4	1.27	(0.91, 1.79)
**Physician consulted**						
No	1121	90.3	120	9.7	Ref.	
Yes	288	77.6	83	22.4	2.49	(1.73, 3.59)
**Hospitalized**						
No	1334	89.1	164	10.9	Ref.	
Yes	75	65.8	39	34.2	4.90	(2.86, 8.39)
**Time interval infection to interview**						
90-120 days	481	85.4	82	14.6	Ref.	
120-180 days	425	87.8	59	12.2	0.74	(0.50, 1.10)
>180 days	503	89.0	62	11.0	0.63	(0.43, 0.94)
**All**	1409	87.4	203	12.6		

[a] Includes Circulatory problems of the heart, stenosis of the coronary arteries, angina pectoris, heart attack, heart failure or heart insufficiency, stroke

[b] Medical conditions associated with weakened immune system or under medications that weaken the immune system

[c] Calculated from weight and height as reported in questionnaire

Table S4 Odds ratios (95% CIs) for reporting a Post-COVID-19 condition in non-hospitalized respondents (N = 1,498) estimated from a multivariable logistic regression model

	Post-COVID-19 condition					
	No		Yes		OR	95% CI
	n	%	n	%		
Sex						
Male	576	93.2	42	6.8	Ref.	
Female	758	86.1	122	13.9	1.58	(1.03, 2.40)
Age (mean, sd)	45.6	18.5	48.8	13.6	1.00	(0.99, 1.02)
Fatigue						
No	404	96.7	14	3.3	Ref.	
Yes	930	86.1	150	13.9	1.80	(0.96, 3.36)
Breathing difficulties						
No	1016	95.0	54	5.0	Ref.	
Yes	318	74.3	110	25.7	4.27	(2.88, 6.32)
Cognitive symptoms						
No	1308	89.7	150	10.3	Ref.	
Yes	26	65.0	14	35.0	3.13	(1.38, 7.12)
Digestive symptoms						
No	1058	91.2	102	8.8	Ref.	
Yes	276	81.7	62	18.3	1.30	(0.86, 1.96)
Fever/Chills						
No	641	90.9	64	9.1	Ref.	
Yes	693	87.4	100	12.6	0.84	(0.56, 1.24)
Head-& Boneaches						
No	426	96.4	16	3.6	Ref.	
Yes	908	86.0	148	14.0	2.28	(1.26, 4.14)
Other respiratory symptoms						
No	421	92.7	33	7.3	Ref.	
Yes	913	87.5	131	12.5	1.14	(0.72, 1.80)
Changes to sense of smell and taste						
No	570	91.5	53	8.5	Ref.	
Yes	764	87.3	111	12.7	1.00	(0.67, 1.50)
Heart & circulatory problems						
No	1301	89.6	151	10.4	Ref.	
Yes	33	71.7	13	28.3	1.29	(0.55, 3.03)
Other Covid-19-related symptoms						
No	1271	89.3	152	10.7	Ref.	
Yes	63	84.0	12	16.0	0.77	(0.36, 1.66)
Other unspecific symptoms						
No	1243	89.6	144	10.4	Ref.	
Yes	91	82.0	20	18.0	1.14	(0.60, 2.16)
High blood pressure or hypertension						
No	1006	89.5	118	10.5	Ref.	
Yes	328	87.7	46	12.3	0.80	(0.50, 1.28)
Other cardiovascular diseases[a]						
No	1251	89.1	153	10.9	Ref.	
Yes	83	88.3	11	11.7	0.60	(0.28, 1.30)
Diabetes mellitus						
No	1276	89.2	155	10.8	Ref.	
Yes	58	86.6	9	13.4	0.71	(0.30, 1.68)
Asthma						
No	1199	90.1	132	9.9	Ref.	
Yes	135	80.8	32	19.2	1.14	(0.63, 2.05)
Chronic lung disease (e.g., COPD)						
No	1238	90.2	134	9.8	Ref.	

Yes	96	76.2	30	23.8	1.76	(0.94, 3.30)
Chronic bronchitis						
No	1279	90.1	141	9.9	Ref.	
Yes	55	70.5	23	29.5	1.60	(0.83, 3.09)
Cancer						
No	1266	89.6	147	10.4	Ref.	
Yes	68	80.0	17	20.0	2.48	(1.11, 5.52)
Currently receiving medical treatment for cancer						
No	1312	89.3	158	10.7	Ref.	
Yes	22	78.6	6	21.4	1.15	(0.31, 4.25)
Weakened immune system[b]						
No	1258	89.8	143	10.2	Ref.	
Yes	76	78.4	21	21.6	1.31	(0.70, 2.46)
Chronic liver disease						
No	1315	89.2	160	10.8	Ref.	
Yes	19	82.6	4	17.4	1.06	(0.28, 4.00)
Adiposity (diagnosed by physician)						
No	1246	89.8	142	10.2	Ref.	
Yes	88	80.0	22	20.0	1.26	(0.62, 2.54)
Education (ISCED)						
Low (ISCED 1, 2)	154	91.1	15	8.9	1,32	(0,65; 2,69)
Medium (ISCED 3, 4)	641	86.4	101	13.6	1,37	(0,90; 2,08)
High (ISCED 5, 6)	503	91.5	47	8.5	Ref.	
Missing	36	97.3	1	2.7	0,94	(0,12; 7,35)
Obese (BMI >30 kg/m²)[c]						
No	1109	90.4	118	9.6	Ref.	
Yes	225	83.0	46	17.0	1.38	(0.82, 2.31)
Smoking status						
Never smoked	798	91.1	78	8.9	Ref.	
Current or ex-smoker	536	86.2	86	13.8	1.26	(0.86, 1.86)
Physician consulted						
No	1046	92.8	81	7.2	Ref.	
Yes	288	77.6	83	22.4	2.22	(1.50, 3.29)
Time interval infection to interview						
90-120 days	457	87.4	66	12.6	Ref.	
120-180 days	404	89.0	50	11.0	0.77	(0.49, 1.21)
>180 days	473	90.8	48	9.2	0.64	(0.41, 1.01)
All	1334	89.1	164	10.9		

[a] Includes Circulatory problems of the heart, stenosis of the coronary arteries, angina pectoris, heart attack, heart failure or heart insufficiency, stroke

[b] Medical conditions associated with weakened immune system or under medications that weaken the immune system

[c] Calculated from weight and height as reported in questionnaire

Fragebogen (CATI)

Teilnahme-ID: __ __ __ __ __

(Vorlage für Telefoninterviews)

Datum des Interviews:

__ __ __ __ __ __ __ __
Tag Monat Jahr

Beginn des Interviews:

_ _ : _ _ Uhr

ID Interviewer/-in:

___ ___

Sprache:

____

Interviewanweisung (nicht vorlesen):
Für diese Befragung werden auch Personen eingeladen, die diesen Fragebogen möglicherweise nicht selbst ausfüllen können (z. B. Kinder, ältere Personen, Menschen mit Behinderungen). Diese Personen können die Fragen mit Hilfe einer anderen Person (z. B. Familienangehörige) beantworten.

Wichtig: *Während des Telefoninterviews (CATI) werden die Fragen 1, 1a und 1b ganz normal vorgelesen und die Antworten notiert. Bei der Dateneingabe ist aber wie folgt vorzugehen:*

Frage 1	*„Nein, ich fülle ihn für eine andere Person aus“*
Frage 1a	*„Für eine andere Person“*
Frage 1b	*Im Freitext-Feld „CATI, InterviewerInnen-ID, Sprache“ eintragen*<br>*Beispiel:* ***CATI , Int.-ID: 01, Sprache: Englisch***

Wenn die interviewte Person für eine andere Person antwortet, bitte diese Person zusätzlich angeben:

Beispiel: ***CATI , Int.-ID: 01, Sprache: Englisch, Für meine Tochter / meinen Sohn***
oder ***CATI , Int.-ID: 05, Sprache: Arabisch, Für meinen Partner / meine Partnerin***

Vielen Dank, dass Sie sich für diese Studie Zeit nehmen. Die Beantwortung wird etwa 15 Minuten dauern.
Sie / die betroffene Person wurde/n für diese Studie ausgewählt, weil das Gesundheitsamt der Stadt Bremen im Zusammenhang mit Ihrer/der betroffenen Person eine Infektion mit dem Virus SARS-CoV2 (im Folgenden Corona-Virus genannt) bzw. eine COVID-19 Erkrankung (im Folgenden auch Corona-Erkrankung genannt) registriert hat. Im Folgenden stellen wir Fragen zum Test auf das Corona-Virus bzw. der Diagnose, zu Beschwerden und zu einigen Lebensumständen.
Mit der Beantwortung dieser Fragen leisten Sie einen wichtigen Beitrag zur Erforschung des Verlaufs der Erkrankung und zur Feststellung des Versorgungsbedarfs bei zukünftigen Patientinnen und Patienten.
Wenn Sie Fragen haben, können Sie diese jederzeit während des Interviews stellen, oder im Anschluss eine/n der verantwortlichen WissenschaftlerInnen kontaktieren. Die Kontaktdaten finden Sie im Einladungsbrief.

Achterstr. 30, 28359 Bremen Telefon 0800-247 72 45 Fax 0421 / 218-56821 E-Mail coverlauf@leibniz-bips.de

1. **Füllen Sie die Befragung für sich selbst oder für eine andere Person aus?**
- ☐ Ja, ich fülle sie für mich selbst aus. *(weiter zu Frage 2)*
- ☐ Nein, ich fülle sie für eine andere Person aus.

1a. Für wen füllen Sie die Befragung aus?
- ☐ Für meinen Partner / meine Partnerin
- ☐ Für meine Tochter / meinen Sohn
- ☐ Für meine Mutter / meinen Vater
- ☐ Für meinen Bruder / meine Schwester
- ☐ Für eine andere Person *(weiter zu Frage 1b)*

1b. Für welche andere Person füllen Sie den Fragebogen aus? *(Freitext)*

____

Sie haben angegeben, dass Sie die Befragung für eine andere Person führen. Bitte denken Sie immer daran, dass bei allen Fragen die Person mit SARS-CoV2-Infektion bzw. COVID-19 Erkrankung gemeint ist.

2. Wann wurden Sie geboren?

Achterstr. 30, 28359 Bremen Telefon 0800-247 72 45 Fax 0421 / 218-56821 E-Mail coverlauf@leibniz-bips.de

Bitte geben Sie nur Monat und Jahr an.

Monat: |__|__| Jahr: |__|__|__|__|

3. Welches Geschlecht haben Sie?

- ☐ Männlich
- ☐ Weiblich
- ☐ Divers
- ☐ Keine Angabe

4. Wie groß sind Sie?

|__|__|__| cm

5. Wie viel wiegen Sie?

|__|__|__| kg

6. Wurde bei Ihnen ein Test auf das Corona-Virus durchgeführt?

- ☐ Ja, einmal
- ☐ Ja, mehrmals
- ☐ Nein *(weiter zu Frage 7)*
- ☐ Weiß nicht *(weiter zu Frage 7)*
- ☐ Keine Angabe *(weiter zu Frage 7)*

6a. War das Testergebnis mindestens einmal positiv, das heißt das Corona-Virus wurde durch einen Test nachgewiesen?

- ☐ Ja
- ☐ Nein *(weiter zu Frage 7)*
- ☐ Weiß nicht *(weiter zu Frage 7)*

6b. Wann wurde der Test auf das Corona-Virus bei Ihnen durchgeführt?
Wenn Sie mehrfach getestet wurden, berichten Sie bitte über den ersten positiven Test.

Bitte Datum hier eingeben: Tag |__|__| Monat |__|__| Jahr |__|__|__|__|

6c. Haben Sie Ihr positives Corona Testergebnis in der Corona-Warn-App geteilt?

- ☐ Ja
- ☐ Nein
- ☐ Ich benutze die Corona-Warn-App nicht.
- ☐ Keine Angabe

(weiter zu Frage 8)

Achterstr. 30, 28359 Bremen Telefon 0800-247 72 45 Fax 0421 / 218-56821 E-Mail coverlauf@leibniz-bips.de

7. Wurde bei Ihnen eine Corona-Erkrankung (COVID-19) allein aufgrund der Krankheits-symptome festgestellt?

- ☐ Ja
- ☐ Nein *(weiter zu Frage 9)*
- ☐ Weiß nicht *(weiter zu Frage 9)*
- ☐ Keine Angabe *(weiter zu Frage 9)*

7a. Wann wurde die Corona-Erkrankung (COVID-19) bei Ihnen festgestellt?

Bitte Datum hier eingeben: Tag |__|__| Monat |__|__| Jahr |__|__|__|__|
(weiter zu Frage 9)

8. Warum wurden Sie auf das Corona-Virus getestet?
Bitte geben Sie alle Antworten an, die zum Zeitpunkt der Testung auf Sie selbst zutrafen.

- ☐ Ich hatte zu diesem Zeitpunkt Symptome, die auf eine Infektion mit dem Corona-Virus hinwiesen.
- ☐ Ich hatte in den 14 Tagen vor Testung Kontakt zu jemandem, bei dem das Corona-Virus im Labor nachgewiesen worden war.
- ☐ Ich hatte in den 14 Tagen vor Testung Kontakt zu jemandem, bei dem der Verdacht auf eine Infektion mit dem Corona-Virus bestand aber kein Testergebnis vorlag.
- ☐ Ich gehöre zur Risikogruppe für schwere Verläufe bei einer Corona-Infektion. *(Die Risikogruppe beinhaltet u.a. die Altersgruppe ≥ 70 Jahre sowie Menschen mit einer Lungen- oder Herz-/Kreislauferkrankung, Zuckerkrankheit, Krebs- oder Autoimmunerkrankung)*
- ☐ Ich war in den 14 Tagen vor Testung aus einem Risikogebiet zurückgekommen.
- ☐ Ich war in den 14 Tagen vor Testung aus dem Ausland zurückgekommen.
- ☐ Ich hatte eine Risikowarnung in der Corona Warn App

Anderer Grund:

____

____

____

____*(Freitext)*

Achterstr. 30, 28359 Bremen Telefon 0800-247 72 45 Fax 0421 / 218-56821 E-Mail coverlauf@leibniz-bips.de

9. Unter welchen Beschwerden (Symptomen) litten Sie zum Zeitpunkt der Corona-Testung / Diagnose und in den ersten Wochen danach?
(Mehrfachnennung möglich)

- ☐ Abgeschlagenheit
- ☐ Atemprobleme
- ☐ Durchfall
- ☐ Fieber
- ☐ Gliederschmerzen
- ☐ Halskratzen
- ☐ Husten
- ☐ Kopfschmerzen
- ☐ Schnupfen
- ☐ Schüttelfrost
- ☐ Übelkeit
- ☐ Geschmacksstörungen
- ☐ Geruchsstörungen
- ☐ Andere Beschwerden *(weiter zu Frage 9a)*
- ☐ Ich hatte keinerlei Krankheitsbeschwerden. *(weiter zu Frage 12)*

(weiter zu Frage 10)

9a. Bitte nennen Sie die anderen Beschwerden (Symptome).

____

____

____

____ *(Freitext)*

Achterstr. 30, 28359 Bremen Telefon 0800-247 72 45 Fax 0421 / 218-56821 E-Mail coverlauf@leibniz-bips.de

10. Waren Sie wegen Ihrer Corona-Infektion in ärztlicher Behandlung?

- ☐ Nein, ich war nicht in ärztlicher Behandlung *(weiter zu Frage 11)*
- ☐ Ja, bei einem/r niedergelassenen/r Arzt/Ärztin. *(weiter zu Frage 10a)*
- ☐ Ja, im Krankenhaus ***(weiter zu Frage 10a: bei ‚Ja' weiter zu Frage 10b, bei ‚Nein' weiter zu Frage 11)***

10a. Wurde bei Ihnen von einem Arzt / einer Ärztin eine Lungenentzündung festgestellt?

- ☐ Ja
- ☐ Nein

(weiter zu Frage 11)

Fragen 10b und 10c nur stellen, wenn Person im Krankenhaus behandelt wurde:

10b. Mussten Sie vorübergehend auf der Intensivstation behandelt werden?

- ☐ Ja
- ☐ Nein *(weiter zu Frage 11)*

10c. Wie viele Tage wurden Sie auf der Intensivstation behandelt?

|__|__|__|Tage

10d. Wurden Sie vorübergehend beatmet?

- ☐ Ja
- ☐ Nein *(weiter zu Frage 11)*

10e. Wie viele Tage wurden Sie beatmet?

|__|__| Tage

Achterstr. 30, 28359 Bremen Telefon 0800-247 72 45 Fax 0421 / 218-56821 E-Mail coverlauf@leibniz-bips.de

11. Wie ist Ihr Gesundheitszustand in Bezug auf die Corona-Infektion aktuell?

- ☐ Ich bin vollständig genesen.
- ☐ Ich habe noch Beschwerden. *(weiter zu Frage 11b)*

11a. Wie lange hatten Sie insgesamt Krankheitssymptome aufgrund der Corona-Infektion?

|__|__| Tage bzw. |__|__| Wochen

(weiter zu Frage 12)

11b. Unter welchen Beschwerden (Symptomen) leiden Sie aktuell im Zusammenhang mit der Corona-Infektion?

(Mehrfachnennung möglich)

- ☐ Abgeschlagenheit
- ☐ Atemprobleme
- ☐ Durchfall
- ☐ Fieber
- ☐ Gliederschmerzen
- ☐ Halskratzen
- ☐ Husten
- ☐ Kopfschmerzen
- ☐ Schnupfen
- ☐ Schüttelfrost
- ☐ Übelkeit
- ☐ Geschmacksstörungen
- ☐ Geruchsstörungen
- ☐ Andere Beschwerden *(weiter zu Frage 11c)*

(weiter zu Frage 12)

11c. Bitte nennen Sie die anderen Beschwerden (Symptome), unter denen Sie aktuell im Zusammenhang mit der Corona-Infektion leiden.

____

____

____*(Freitext)*

Achterstr. 30, 28359 Bremen Telefon 0800-247 72 45 Fax 0421 / 218-56821 E-Mail coverlauf@leibniz-bips.de

12. Hatten Sie seit Oktober 2019 eine andere Atemwegserkrankung (zum Beispiel Husten, Schnupfen, Halsschmerzen) mit oder ohne Fieber?

- ☐ Ja
- ☐ Nein *(weiter zu Frage 13)*

12a. Bitte geben Sie an, ob Sie während der anderen Atemwegserkrankung folgende Beschwerden hatten.

- ☐ Husten
- ☐ Halsschmerzen
- ☐ Fieber

13. Haben Sie sich seit Oktober 2019 gegen die Grippe (Influenza) impfen lassen?

- ☐ Ja
- ☐ Nein *(weiter zu Frage 14)*

13a. Wann haben Sie sich gegen Grippe (Influenza) impfen lassen?

Bitte geben Sie nur Monat und Jahr an.

Monat: |__|__| Jahr: |__|__|__|__|

- ☐ Ich kann mich nicht an den genauen Zeitpunkt erinnern.

Achterstr. 30, 28359 Bremen Telefon 0800-247 72 45 Fax 0421 / 218-56821 E-Mail coverlauf@leibniz-bips.de

14. Die folgenden Fragen beziehen sich auf Vorerkrankungen, die schon vor der Corona-Pandemie bei Ihnen bekannt waren.
(Mehrfachnennung möglich)

	ja	unsicher	nein
Ist bei Ihnen jemals ein erhöhter oder zu hoher Blutdruck festgestellt worden?	☐	☐	☐
Wurde bei Ihnen jemals von einem Arzt/ einer Ärztin eine Durchblutungsstörung am Herzen, eine Verengung der Herzkranzgefäße oder eine Angina pectoris festgestellt?	☐	☐	☐
Hat ein Arzt / eine Ärztin bei Ihnen schon einmal einen Herzinfarkt festgestellt?	☐	☐	☐
Hat ein Arzt / eine Ärztin jemals bei Ihnen eine Herzschwäche oder Herzinsuffizienz festgestellt?	☐	☐	☐
Hat ein Arzt / eine Ärztin bei Ihnen schon einmal einen Schlaganfall festgestellt?	☐	☐	☐
Hat ein Arzt / eine Ärztin jemals bei Ihnen eine Zuckerkrankheit bzw. Diabetes mellitus festgestellt?	☐	☐	☐
Wurde bei Ihnen jemals von einem Arzt / einer Ärztin Asthma festgestellt?	☐	☐	☐
Wurde bei Ihnen jemals von einem Arzt / einer Ärztin eine chronische Lungenkrankheit (z. B. COPD, Asthma) festgestellt?<br>→ Wenn ja, welche andere Lungenerkrankung liegt vor_____________	☐	☐	☐
Hat ein Arzt / eine Ärztin bei Ihnen jemals eine chronische Bronchitis festgestellt?	☐	☐	☐
Hat ein Arzt / eine Ärztin bei Ihnen jemals eine Krebserkrankung festgestellt?	☐	☐	☐
Sind Sie zurzeit wegen der Krebserkrankung in ärztlicher Behandlung?	☐	☐	☐
Haben Sie eine Erkrankung, die mit einer Immunschwäche einhergeht oder die Einnahme von Medikamenten erforderlich macht, welche die Immunabwehr schwächen, wie z. B. Cortison?	☐	☐	☐
Hat ein Arzt / eine Ärztin bei Ihnen jemals eine chronische Leber-erkrankung festgestellt?	☐	☐	☐
Hat ein Arzt / eine Ärztin bei Ihnen jemals Adipositas festgestellt?	☐	☐	☐

Achterstr. 30, 28359 Bremen Telefon 0800-247 72 45 Fax 0421 / 218-56821 E-Mail coverlauf@leibniz-bips.de

Im Folgenden stellen wir Fragen zum Rauchverhalten, zur Lebenssituation und zur Schul- und Berufsbildung. Die Angaben zum Rauchverhalten sind wichtig, weil Rauchen den Verlauf von Atemwegserkrankungen beeinflussen kann. Die Informationen zur Wohnsituation und zur Bildung dienen zur Einschätzung der Kontaktsituation in Privat- und Berufsleben und zur Erkennung von möglichen Infektionsherden.

15. Haben Sie in Ihrem Leben jemals über einen Zeitraum von mehr als 6 Monaten regelmäßig geraucht?

Regelmäßig bedeutet: eine Zigarette pro Tag oder mindestens fünf Zigaretten pro Woche oder mindestens eine Packung Zigaretten pro Monat, zwei Zigarren pro Woche oder zwei Pfeifen pro Woche.

Wenn mindestens eins zutrifft, kreuzen Sie ‚Ja' an.

- ☐ Ja *(weiter zu Frage 16 und danach weiter zu Frage 17)*
- ☐ Nein

16. Benutzen Sie derzeit eine elektrische Zigarette (E-Zigarette) oder ein ähnliches Produkt (wie zum Beispiel E-Shisha, E-Zigarre oder E-Pfeife)?

- ☐ Ja
- ☐ Nein

(Weiter mit Frage 18)

Frage nur stellen, wenn bei Frage 15 ‚Ja' angekreuzt wurde:

17. Wann haben Sie angefangen, regelmäßig Tabak zu rauchen? Sie können entweder ihr damaliges Alter angeben oder die Jahreszahl.

Ich war |__|__|Jahre alt **oder** im Jahr |__|__|__|__|.

17a. Rauchen Sie zurzeit täglich, gelegentlich oder überhaupt keinen Tabak?

- ☐ Ja, täglich *(weiter zu Frage 17b)*
- ☐ Ja, gelegentlich *(weiter zu Frage 17c)*
- ☐ Nein, überhaupt nicht *(weiter zu Frage 17d und 17e)*

17b. Wie viele Zigaretten, Zigarren oder Pfeifen rauchen Sie zurzeit täglich?

Anzahl Zigaretten: |__|__| pro **Tag**

Anzahl Zigarren: |__|__| pro **Tag**

Anzahl Pfeifen: |__|__| pro **Tag**

(weiter zu Frage 18)

Achterstr. 30, 28359 Bremen Telefon 0800-247 72 45 Fax 0421 / 218-56821 E-Mail coverlauf@leibniz-bips.de

17c. Wie viele Zigaretten, Zigarren oder Pfeifen rauchen Sie zurzeit gelegentlich?

Anzahl Zigaretten: |__|__| pro **Woche**

Anzahl Zigarren: |__|__| pro **Woche**

Anzahl Pfeifen: |__|__| pro **Woche**
(weiter zu Frage 18)

17d. Wann haben Sie aufgehört zu rauchen? Sie können entweder ihr damaliges Alter angeben oder die Jahreszahl.

Ich war |__|__|Jahre alt **bzw.** ich habe', 'sie hat' im Jahr |__|__|__|__|aufgehört.

17e. Wie viel haben Sie früher geraucht?
Bitte geben Sie die Anzahl Zigaretten, Zigarren und Pfeifen pro Tag oder pro Woche an. Wenn Sie gelegentlich Zigaretten rauchen, geben Sie die Anzahl pro Woche an.

Anzahl Zigaretten: |__|__| pro **Tag** **oder** Anzahl Zigaretten: |__|__| pro **Woche**

Anzahl Zigarren: |__|__| pro **Tag** **oder** Anzahl Zigarren: |__|__| pro **Woche**

Anzahl Pfeifen: |__|__| pro **Tag** **oder** Anzahl Pfeifen: |__|__| pro **Woche**

Im Folgenden stellen wir Ihnen Fragen zu Ihrer Wohnsituation.

18. Wie wohnen Sie?
- ☐ In einer Wohnung (zur Miete oder Eigentum)
- ☐ In einem Einfamilienhaus oder Reihenhaus (zur Miete oder Eigentum)
- ☐ Im „betreuten Wohnen“
- ☐ In einem Seniorenheim *(weiter zu Frage 20)*
- ☐ In einem Pflegeheim *(weiter zu Frage 20)*
- ☐ In einer Unterkunft für Geflüchtete *(weiter zu Frage 20)*
- ☐ Weiß nicht
- ☐ Keine Angabe

19. Wie viele Personen leben in Ihrem Haushalt / Ihrer Wohngemeinschaft, Sie selbst mitgerechnet?

Anzahl |__|__| *(nicht mehr als 16 möglich)*
(Wenn Antwort 1 (=die Person wohnt alleine) → weiter zu Frage 21)

Achterstr. 30, 28359 Bremen Telefon 0800-247 72 45 Fax 0421 / 218-56821 E-Mail coverlauf@leibniz-bips.de

19a. Waren oder sind andere Personen aus Ihrem Haushalt / Ihrer Wohngemeinschaft an Corona (COVID-19) erkrankt oder hatten einen positiven Corona-Test?

- ☐ Ja
- ☐ Nein *(weiter zu Frage 21)*

19b. Wie viele Personen aus Ihrem Haushalt / Ihrer Wohngemeinschaft waren oder sind an Corona (COVID-19) erkrankt oder hatten einen positiven Corona-Test, Sie selbst nicht mitgerechnet?

Anzahl |__|__| *(nicht mehr als 15 möglich)*

19c. Welche Personen aus Ihrem Haushalt / Ihrer Wohngemeinschaft waren oder sind an der Corona-Erkrankung (COVID-19) erkrankt oder hatten einen positiven Corona-Test?
Diese Angaben sind notwendig für die Untersuchung von Infektionsherden.

1. Haushaltsmitglied	Wann wurde diese Person geboren?<br>*Bitte geben Sie nur Monat und Jahr an.*	Wurde ein Corona-Test bei dieser Person durchgeführt?
☐ Partner / Partnerin<br>☐ Tochter / Sohn<br>☐ Mutter / Vater<br>☐ Bruder / Schwester<br>☐ Andere Person	Monat: |__|__| Jahr: |__|__|__|__|	☐ Ja<br>☐ Nein
2. Haushaltsmitglied	Wann wurde diese Person geboren?<br>*Bitte geben Sie nur Monat und Jahr an.*	Wurde ein Corona-Test bei dieser Person durchgeführt?
☐ Partner / Partnerin<br>☐ Tochter / Sohn<br>☐ Mutter / Vater<br>☐ Bruder / Schwester<br>☐ Andere Person	Monat: |__|__| Jahr: |__|__|__|__|	☐ Ja<br>☐ Nein
3. Haushaltsmitglied	Wann wurde diese Person geboren?<br>*Bitte geben Sie nur Monat und Jahr an.*	Wurde ein Corona-Test bei dieser Person durchgeführt?
☐ Partner / Partnerin<br>☐ Tochter / Sohn<br>☐ Mutter / Vater<br>☐ Bruder / Schwester<br>☐ Andere Person	Monat: |__|__| Jahr: |__|__|__|__|	☐ Ja<br>☐ Nein

Für weitere Personen „Vorlage Haushaltsmitglieder“ am Ende des Dokuments nutzen.

(weiter zu Frage 21)

Achterstr. 30, 28359 Bremen Telefon 0800-247 72 45 Fax 0421 / 218-56821 E-Mail coverlauf@leibniz-bips.de

20. Schlafen mehrere Personen in dem Raum, in dem Sie selbst schlafen?

- ☐ Ja *(weiter zu Frage 20a und 20b)*
- ☐ Nein *(weiter zu Frage 21)*

20a. Wie viele Personen schlafen mit Ihnen in einem Raum, Sie selbst <u>nicht</u> mitgerechnet?

Anzahl |__|__|

20b. Waren oder sind andere Personen, mit denen Sie in einem Raum schlafen Corona (COVID-19) erkrankt oder hatten einen positiven Corona-Test?

- ☐ Ja
- ☐ Nein

21. Bitte geben Sie die Postleitzahl Ihrer Wohnadresse an:

|__|__|__|__|__|

22. Welchen höchsten allgemeinbildenden Schulabschluss haben Sie? *(nur eine Antwort möglich)*

- ☐ Ich bin noch Schüler/-in an einer allgemeinbildenden Schule.
- ☐ Ich bin ohne Hauptschulabschluss [Volksschulabschluss] von der Schule abgegangen.
- ☐ Hauptschulabschluss [Volksschulabschluss]
- ☐ Realschulabschluss [Mittlere Reife]
- ☐ Polytechnische Oberschule der DDR mit Abschluss der 8. oder 9. Klasse
- ☐ Polytechnische Oberschule der DDR mit Abschluss der 10. Klasse
- ☐ Fachhochschulreife, Abschluss einer Fachoberschule
- ☐ Allgemeine oder fachgebundene Hochschulreife/Abitur [Gymnasium bzw. EOS, auch EOS mit Lehre]
- ☐ Abitur über den zweiten Bildungsweg nachgeholt
- ☐ Einen anderen Schulabschluss *(weiter zu Frage 22a)*
- ☐ Weiß nicht
- ☐ Keine Angabe

(weiter zu Frage 23)

Achterstr. 30, 28359 Bremen Telefon 0800-247 72 45 Fax 0421 / 218-56821 E-Mail coverlauf@leibniz-bips.de

22a. Sie haben angegeben, dass Sie einen anderen Schulabschluss haben. Bitte nennen Sie diesen Schulabschluss.

____ *(Freitext)*

23. Welche beruflichen Ausbildungsabschlüsse haben Sie?
Falls mehrere Antworten zutreffen, geben Sie bitte den höchsten Berufsabschluss an.

- ☐ Noch in beruflicher Ausbildung [Berufsvorbereitungsjahr, Auszubildende(r), Praktikant/-in, Student/-in]
- ☐ Schüler/-in und besuche eine berufsorientierte Aufbau-, Fachschule o. ä.
- ☐ Keinen beruflichen Abschluss und nicht in beruflicher Ausbildung
- ☐ Beruflich-betriebliche Berufsausbildung [Lehre] abgeschlossen
- ☐ Beruflich-schulische Ausbildung abgeschlossen [Berufsfachschule, Handelsschule, Vorbereitungsdienst für den mittleren Dienst in der öffentlichen Verwaltung]
- ☐ Ausbildung an einer Fachschule der DDR abgeschlossen
- ☐ Ausbildung an einer Fach-, Meister- oder Technikerschule, Berufs- oder Fachakademie abgeschlossen
- ☐ Bachelor an einer (Fach-)Hochschule abgeschlossen
- ☐ Fachhochschulabschluss [z. B. Diplom, Master]
- ☐ Universitätsabschluss [z. B. Diplom, Magister, Staatsexamen, Master]
- ☐ Promotion
- ☐ Einen anderen beruflichen Abschluss *(weiter zu Frage 23a)*
- ☐ Weiß nicht
- ☐ Keine Angabe

(weiter zu Frage 24)

23a. Sie haben angegeben, dass Sie einen anderen beruflichen Abschluss haben. Bitte nennen Sie diesen beruflichen Abschluss.

____ *(Freitext)*

Achterstr. 30, 28359 Bremen Telefon 0800-247 72 45 Fax 0421 / 218-56821 E-Mail coverlauf@leibniz-bips.de

24. Sind Sie zurzeit erwerbstätig?
Falls mehrere Antworten zutreffen, wählen Sie bitte die Antwort, die am besten zutrifft.

- ☐ Vollzeit erwerbstätig *(weiter zu Frage 25)*
- ☐ Teilzeit erwerbstätig *(weiter zu Frage 25)*
- ☐ Altersteilzeit [unabhängig davon, ob in der Arbeits- oder Freistellungsphase befindlich] *(weiter zu Frage 25)*
- ☐ Geringfügig erwerbstätig, 450 Euro- oder Mini-Job *(weiter zu Frage 25)*
- ☐ „Ein-Euro-Job" [bei Bezug von Arbeitslosengeld II] *(weiter zu Frage 25)*
- ☐ Gelegentlich oder unregelmäßig beschäftigt
- ☐ In einer beruflichen Ausbildung/Lehre *(weiter zu Frage 25)*
- ☐ In Umschulung
- ☐ Bundesfreiwilligendienst, Freiwilliges Soziales/Ökologisches Jahr
- ☐ Mutterschafts-, Erziehungsurlaub, Elternzeit oder sonstige Beurlaubung
- ☐ Nicht erwerbstätig [einschließlich: Schüler/-innen oder Studierende, die nicht gegen Geld arbeiten, Arbeitslose, Vorruheständler/-innen, Rentner/-innen ohne Nebenverdienst]
- ☐ Weiß nicht
- ☐ Keine Angabe

25. Welchen Beruf üben Sie derzeit aus?

____ *(Freitext)*

26. Sollten Sie weitere Kommentare oder Anmerkungen haben, haben Sie jetzt die Möglichkeit, uns das mitzuteilen. Bitte geben Sie keine Namen, Adressen oder andere persönliche Informationen an. Bitte beachten Sie, dass es uns aus Datenschutzgründen nicht möglich ist, Ihnen auf Ihre Kommentare oder Fragen zu antworten.
Wenn Sie mehr als einen <u>positiven</u> Test hatten und der Abstand zwischen diesen Tests mehr als 2 Wochen betrug, tragen Sie hier bitte für alle Tests das Datum ein. Falls bekannt, auch den Grund für die Mehrfachtestung. Bitte geben Sie nur PCR-Tests an (d.h. keine Antigen-Schnelltests oder Blutuntersuchungen).

____

____

____

____ *(Freitext)*

Vielen Dank für Ihre Unterstützung! Mit Ihrer Teilnahme an der Befragung haben Sie einen wichtigen Beitrag zur Erforschung der Corona-Erkrankung (COVID-19) geleistet.

Achterstr. 30, 28359 Bremen Telefon 0800-247 72 45 Fax 0421 / 218-56821 E-Mail coverlauf@leibniz-bips.de

„Vorlage Haushaltsmitglieder“

Ergänzung zu Frage 19c, ab 4. Haushaltsmitglied

4. Haushaltsmitglied	Wann wurde diese Person geboren?<br>*Bitte geben Sie nur Monat und Jahr an.*	Wurde ein Corona-Test bei dieser Person durchgeführt?
☐ Partner / Partnerin<br>☐ Tochter / Sohn<br>☐ Mutter / Vater<br>☐ Bruder / Schwester<br>☐ Andere Person	Monat: |__|__| Jahr: |__|__|__|__|	☐ Ja<br>☐ Nein
5. Haushaltsmitglied	Wann wurde diese Person geboren?<br>*Bitte geben Sie nur Monat und Jahr an.*	Wurde ein Corona-Test bei dieser Person durchgeführt?
☐ Partner / Partnerin<br>☐ Tochter / Sohn<br>☐ Mutter / Vater<br>☐ Bruder / Schwester<br>☐ Andere Person	Monat: |__|__| Jahr: |__|__|__|__|	☐ Ja<br>☐ Nein
6. Haushaltsmitglied	Wann wurde diese Person geboren?<br>*Bitte geben Sie nur Monat und Jahr an.*	Wurde ein Corona-Test bei dieser Person durchgeführt?
☐ Partner / Partnerin<br>☐ Tochter / Sohn<br>☐ Mutter / Vater<br>☐ Bruder / Schwester<br>☐ Andere Person	Monat: |__|__| Jahr: |__|__|__|__|	☐ Ja<br>☐ Nein
7. Haushaltsmitglied	Wann wurde diese Person geboren?<br>*Bitte geben Sie nur Monat und Jahr an.*	Wurde ein Corona-Test bei dieser Person durchgeführt?
☐ Partner / Partnerin<br>☐ Tochter / Sohn<br>☐ Mutter / Vater<br>☐ Bruder / Schwester<br>☐ Andere Person	Monat: |__|__| Jahr: |__|__|__|__|	☐ Ja<br>☐ Nein
8. Haushaltsmitglied	Wann wurde diese Person geboren?<br>*Bitte geben Sie nur Monat und Jahr an.*	Wurde ein Corona-Test bei dieser Person durchgeführt?
☐ Partner / Partnerin<br>☐ Tochter / Sohn<br>☐ Mutter / Vater<br>☐ Bruder / Schwester<br>☐ Andere Person	Monat: |__|__| Jahr: |__|__|__|__|	☐ Ja<br>☐ Nein

Achterstr. 30, 28359 Bremen Telefon 0800-247 72 45 Fax 0421 / 218-56821 E-Mail coverlauf@leibniz-bips.de

9. Haushaltsmitglied	Wann wurde diese Person geboren?<br>*Bitte geben Sie nur Monat und Jahr an.*	Wurde ein Corona-Test bei dieser Person durchgeführt?
☐ Partner / Partnerin<br>☐ Tochter / Sohn<br>☐ Mutter / Vater<br>☐ Bruder / Schwester<br>☐ Andere Person	Monat: |__|__| Jahr: |__|__|__|__|	☐ Ja<br>☐ Nein
10. Haushaltsmitglied	Wann wurde diese Person geboren?<br>*Bitte geben Sie nur Monat und Jahr an.*	Wurde ein Corona-Test bei dieser Person durchgeführt?
☐ Partner / Partnerin<br>☐ Tochter / Sohn<br>☐ Mutter / Vater<br>☐ Bruder / Schwester<br>☐ Andere Person	Monat: |__|__| Jahr: |__|__|__|__|	☐ Ja<br>☐ Nein
11. Haushaltsmitglied	Wann wurde diese Person geboren?<br>*Bitte geben Sie nur Monat und Jahr an.*	Wurde ein Corona-Test bei dieser Person durchgeführt?
☐ Partner / Partnerin<br>☐ Tochter / Sohn<br>☐ Mutter / Vater<br>☐ Bruder / Schwester<br>☐ Andere Person	Monat: |__|__| Jahr: |__|__|__|__|	☐ Ja<br>☐ Nein
12. Haushaltsmitglied	Wann wurde diese Person geboren?<br>*Bitte geben Sie nur Monat und Jahr an.*	Wurde ein Corona-Test bei dieser Person durchgeführt?
☐ Partner / Partnerin<br>☐ Tochter / Sohn<br>☐ Mutter / Vater<br>☐ Bruder / Schwester<br>☐ Andere Person	Monat: |__|__| Jahr: |__|__|__|__|	☐ Ja<br>☐ Nein

Achterstr. 30, 28359 Bremen Telefon 0800-247 72 45 Fax 0421 / 218-56821 E-Mail coverlauf@leibniz-bips.de

Questionnaire CoVerlauf (CATI)

(Template for telephone interview)

Participant-ID: __ __ __ __ __

Date of interview:

__ __ __ __ __ __ __ __
day month year

Beginning of interview:

_ _ : _ _ a.m. / p.m. (please mark the daytime)

ID of the interviewer:

___ ___

Language:

____

Note (do not read out):
This survey is also addressed to people who may not be able to fill out this questionnaire on their own (e.g., children, the elderly, people with disabilities). These people may answer the questions with the assistance of another person (e.g., family members).
Important: *During interviews by phone (CATI) questions 1, 1a, and 1b are read out as written and answers are noted. When entering the data into the online database, however, please proceed as follows:*

Question 1	*"No, I am filling it out for another person."*
Question 1a	*"For another person"*
Question 1b	*Enter CATI in the free text field*
	Example: ***CATI , Int.-ID: 01, Sprache: Englisch***

If the interviewed person does take part for another person, please also provide the answer the person gave to Question 1a

Example: ***CATI , Int.-ID: 01, Sprache: Englisch, Für meine Tochter / meinen Sohn***
or ***CATI , Int.-ID: 05, Sprache: Arabisch, Für meinen Partner / meine Partnerin***

Thank you for taking the time to participate in this study. Answering the questions will take about 15 minutes.
You were selected for this study because the public health department of the city of Bremen has registered an infection with the SARS-CoV2 virus (hereinafter referred to as Corona virus) or with COVID-19 disease (hereinafter also referred to as Corona disease) for your person.
In the following, we will ask you questions about the test for the Corona virus or your diagnosis, about your symptoms, and about your living conditions.
By answering these questions, you are making an important contribution to the research on the course of the disease and you will help us determine what is needed in terms of care for future patients.
If you have questions, please feel free to ask at any time during the interview, or to contact the responsible scientists after the interview. You'll find their contact information in the invitation letter.

Achterstr. 30, 28359 Bremen Telefon 0800-247 72 45 Fax 0421 / 218-56821 E-Mail coverlauf@leibniz-bips.de

1. Are you filling out the questionnaire for yourself or for another person?

- ☐ Yes, I am filling it out for myself. *(continue with question 2)*
- ☐ No, I am filling it out for another person.

1a. For whom are you filling out the questionnaire?

- ☐ For my partner
- ☐ For my daughter / my son
- ☐ For my mother / my father
- ☐ For my brother / my sister
- ☐ For another person *(continue with question 1b)*

1b. For which other person are you filling out the questionnaire? *(free text)*

____

You indicated that you are filling the questionnaire for another person. Please note, whenever the term "person concerned" is used, it refers to the person with the SARS-CoV2 infection with virus or with the COVID-19 disease.

Achterstr. 30, 28359 Bremen Telefon 0800-247 72 45 Fax 0421 / 218-56821 E-Mail coverlauf@leibniz-bips.de

2. When were you born?
Please enter month and year only.

Month: |__|__| Year: |__|__|__|__|

3. What sex are you?

- ☐ Male
- ☐ Female
- ☐ Diverse
- ☐ No answer

4. How tall are you?

|__|__|__| cm

5. How much do you weigh?

|__|__|__| kg

6. Have you been tested for the Corona virus?

- ☐ Yes, once
- ☐ Yes, multiple times
- ☐ No *(continue with question 7)*
- ☐ Don't know *(continue with question 7)*
- ☐ No answer *(continue with question 7)*

6a. Was the test result at least once positive, meaning the Corona virus was confirmed by a test?

- ☐ Yes
- ☐ No *(continue with question 7)*
- ☐ Don't know *(continue with question 7)*

6b. When did you have the test for the Corona virus?
If tested multiple times, please report on the first positive test.

Please enter date here: day |__|__| month |__|__| year |__|__|__|__|

6c. Did you share the positive result in the Corona-Warn-App?

- ☐ Yes
- ☐ No
- ☐ I do not use the Corona-Warn-App.
- ☐ No answer

(continue with question 8)

Achterstr. 30, 28359 Bremen Telefon 0800-247 72 45 Fax 0421 / 218-56821 E-Mail coverlauf@leibniz-bips.de

7. **Have you been diagnosed with the Corona disease (COVID 19) solely based on your symptoms?**

- ☐ Yes
- ☐ No *(continue with question 9)*
- ☐ Don't know *(continue with question 9)*
- ☐ No answer *(continue with question 9)*

7a. When were you diagnosed with the Corona disease (COVID-19)?

Please enter date here: day |__|__| month |__|__| year |__|__|__|__|
(continue with question 9)

8. Why were you tested for the Corona virus?
Please select all answers that applied at the time of the test.

- ☐ At that time, I had symptoms suggesting an infection with the Corona virus.
- ☐ In the 14 days before testing, I was in contact with someone for whom the Corona virus had been confirmed in a laboratory.
- ☐ In the 14 days before testing, I was in contact with someone for whom there was suspicion of an infection with the Corona virus but there was no test result.
- ☐ I belong to the risk group for a severe course of a Corona infection.
  (This risk group includes, among others, the age group ≥ 70 years and people with a lung or cardiovascular disease, diabetes, cancer or autoimmune disease)
- ☐ In the 14 days before testing, I had returned from a risk area.
- ☐ In the 14 days before testing, I had returned from abroad.
- ☐ I had a risk message in the Corona Warn App

Other reason:

____

____

____

____*(free text)*

Achterstr. 30, 28359 Bremen Telefon 0800-247 72 45 Fax 0421 / 218-56821 E-Mail coverlauf@leibniz-bips.de

9. **Which problems (symptoms) did you have at the time of the Corona test/diagnosis and in the first weeks thereafter?**
(Multiple answers possible)

- ☐ Fatigue
- ☐ Respiratory problems
- ☐ Diarrhea
- ☐ Fever
- ☐ Joint pain
- ☐ Scratchy throat
- ☐ Cough
- ☐ Headache
- ☐ Sniffles
- ☐ Chills
- ☐ Nausea
- ☐ Loss of taste
- ☐ Loss of smell
- ☐ Other problems *(continue with question 9a)*
- ☐ I did not have any symptoms at all. *(continue with question 12)*

(continue with question 10)

9a. Please name the other problems (symptoms).

____

____

____

____*(free text)*

Achterstr. 30, 28359 Bremen Telefon 0800-247 72 45 Fax 0421 / 218-56821 E-Mail coverlauf@leibniz-bips.de

10. **Did you receive medical treatment because of the Corona infection?**

- ☐ No, I did not receive medical treatment *(continue with question 11)*
- ☐ Yes, from a general practitioner. *(continue with question 10a)*
- ☐ Yes, in the hospital. ***(continue with question 10a: if ‚Yes' continue with question 10b, if ‚No' continue with question 11)***

10a. Have you been diagnosed by a doctor with pneumonia?

- ☐ Yes
- ☐ No

(continue with question 11)

Only ask questions 10b and 10c if person was treated in a hospital:

10b. Did you have to be treated temporarily in an intensive care unit?

- ☐ Yes
- ☐ No *(continue with question 11)*

10c. How many days were you treated in the intensive care unit?

|__|__|__|days

10d. Were you temporarily ventilated?

- ☐ Yes
- ☐ No *(continue with question 11)*

10e. How many days were you ventilated?

|__|__| days

Achterstr. 30, 28359 Bremen Telefon 0800-247 72 45 Fax 0421 / 218-56821 E-Mail coverlauf@leibniz-bips.de

11. How is your current state of health regarding the Corona infection?

- ☐ I am fully recovered.
- ☐ I still have problems. *continue with question 11b)*

11a. In total, for how long did you have symptoms because of the Corona infection?

|__|__| days **or** |__|__| weeks
(continue with question 12)

11b. Which problems (symptoms) related to the Corona infection are you currently experiencing?
(Multiple answers possible)

- ☐ Fatigue
- ☐ Respiratory problems
- ☐ Diarrhea
- ☐ Fever
- ☐ Joint pain
- ☐ Scratchy throat
- ☐ Cough
- ☐ Headache
- ☐ Sniffles
- ☐ Chills
- ☐ Nausea
- ☐ Loss of taste
- ☐ Loss of smell
- ☐ Other problems *(continue with question 11c)*

(continue with question 12)

11c. Please name the other current problems (symptoms) related to the Corona infection.

____

____

____*(free text)*

Achterstr. 30, 28359 Bremen Telefon 0800-247 72 45 Fax 0421 / 218-56821 E-Mail coverlauf@leibniz-bips.de

12. Did you have another respiratory illness since October 2019 (for example, cough, sniffles, sore throat with or without a fever)?

- ☐ Yes
- ☐ No *(continue with question 13)*

12a. Please indicate whether you experienced the following problems during the other respiratory illness:

- ☐ Cough
- ☐ Sore throat
- ☐ Fever

13. Have you been vaccinated against the flu (influenza) since October 2019?

- ☐ Yes
- ☐ No *(continue with question 14)*

13a. When were you vaccinated against the flu (influenza)?

Please enter month and year only.

Month: |__|__| Year: |__|__|__|__|

- ☐ I do not remember the exact date.

Achterstr. 30, 28359 Bremen Telefon 0800-247 72 45 Fax 0421 / 218-56821 E-Mail coverlauf@leibniz-bips.de

14. The following questions are related to your pre-existing conditions were already known before the Corona pandemic. *(Multiple answers possible)*			
	**Yes**	**Uncertain**	**No**
Have you ever been diagnosed with high blood pressure or hypertension?	☐	☐	☐
Have you ever been diagnosed by a doctor with circulatory problems of the heart, stenosis of the coronary arteries or angina pectoris?	☐	☐	☐
Has a doctor ever diagnosed you with a heart attack?	☐	☐	☐
Has a doctor ever diagnosed you with heart failure or heart insufficiency?	☐	☐	☐
Has a doctor ever diagnosed you with a stroke?	☐	☐	☐
Has a doctor ever diagnosed you with diabetes mellitus?	☐	☐	☐
Have you ever been diagnosed by a doctor with asthma?	☐	☐	☐
Have you ever been diagnosed by a doctor with chronic lung disease (e.g., COPD, asthma?<br>→ If so, what other lung disease is present?<br>____	☐	☐	☐
Has a doctor ever diagnosed you with chronic bronchitis?	☐	☐	☐
Has a doctor ever diagnosed you with cancer?	☐	☐	☐
Are you currently receiving medical treatment for cancer?	☐	☐	☐
Do you have a medical condition associated with a weakened immune system or requiring it to take medications that weaken the immune system, e.g., cortisone?	☐	☐	☐
Has a doctor ever diagnosed you with chronic liver disease?	☐	☐	☐
Has a doctor ever diagnosed you with adiposity?	☐	☐	☐

Achterstr. 30, 28359 Bremen Telefon 0800-247 72 45 Fax 0421 / 218-56821 E-Mail coverlauf@leibniz-bips.de

In the following, we will ask you questions about your smoking behavior, your living conditions, your schooling, and your vocational training. The information on your smoking behavior is important because smoking can influence the course of respiratory diseases. The information on your living conditions and education will serve to assess your contact situation in your private and professional life and to identify possible sources of infection.

15. Have you ever smoked regularly for a period longer than six months?

Regularly means: One cigarette per day or at least five cigarettes per week or at least one package of cigarettes per month, two cigars per week or two pipes per week.

If at least one applies, select ‚Yes'

- ☐ Yes *(continue with question 16 and after that continue with question 17)*
- ☐ No

16. Do you currently use an electric cigarette (e-cigarette) or a similar product (for example, e-shisha, e-cigar or e-pipe)?

- ☐ Yes
- ☐ No

(continue with question 18)

Only display questions 17 to 17e if question 15 was answered ‚Yes':

17. When did you start smoking tobacco regularly? You might either answer with your age at the time or the year.

I was |__|__| years old **or** in the year |__|__|__|__|.

17a. Do you currently smoke tobacco daily, occasionally or not at all?

- ☐ Yes, daily *(continue with question 17b)*
- ☐ Yes, occasionally *continue with question 17c)*
- ☐ No, not at all *(continue with question 17d and 17e)*

17b. How many cigarettes, cigars or pipes do you currently smoke?

Number of cigarettes: |__|__| per **day**

Number of cigars: |__|__| per **day**

Number of pipes: |__|__| per **day**

(continue with question 18)

Achterstr. 30, 28359 Bremen Telefon 0800-247 72 45 Fax 0421 / 218-56821 E-Mail coverlauf@leibniz-bips.de

17c. How many cigarettes, cigars or pipes do you currently smoke?

Number of cigarettes: |__|__| per **week**

Number of cigars: |__|__| per **week**

Number of pipes: |__|__| per **week**
(continue with question 18)

17d. When did you quit smoking? You might either answer with your age at the time or the year.

I was |__|__| years old **<u>or</u>** I quit in the year |__|__|__|__|.

17e. How much did you smoke in the past?
Please indicate the number of cigarettes, cigars, and pipes per day or per week. If you occasionally smoke cigarettes, please indicate the number per week.

Number of cigarettes: |__|__| per **day** **<u>or</u>** number of cigarettes: |__|__| per **week**

Number of cigars: |__|__| per **day** **<u>or</u>** number of cigars: |__|__| per **week**

Number of pipes: |__|__| per **day** **<u>or</u>** number of pipes: |__|__| per **week**

In the following we will ask you questions about your living situation.

18. Where do you live?

- ☐ In an apartment (rented or owned)
- ☐ In a single family or row house (rented or owned)
- ☐ In assisted living
- ☐ In a retirement home *(continue with question 20)*
- ☐ In a nursing home *(continue with question 20)*
- ☐ In refugee housing *(continue with question 20)*
- ☐ Don’t know
- ☐ No answer

19. How many people live in your household / shared housing, yourself included?

Number |__|__| *(no more than 16 possible)*
(If answer 1 (=the person lives alone) → continue with question 21)

Achterstr. 30, 28359 Bremen Telefon 0800-247 72 45 Fax 0421 / 218-56821 E-Mail coverlauf@leibniz-bips.de

19a. Did or do other persons from your household / shared housing have Corona (COVID-19) or did they have a positive Corona test?

- ☐ Yes
- ☐ No *(continue with question 21)*

19b. How many persons from your household / shared housing had or have Corona (COVID-19) or had a positive Corona test, yourself <u>not</u> included?

Number |__|__| *(no more than 15 possible)*

19c. Which persons from your household / shared housing had or have Corona (COVID-19) or had a positive Corona test?

This information is necessary for the investigation of sources of infection.

1. Member of household	When was this person born?<br>*Please enter month and year only.*	Did this person have a corona test?
☐ Partner<br>☐ Daughter / son<br>☐ Mother / father<br>☐ Brother / sister<br>☐ Other person	Month: |__|__| Year: |__|__|__|__|	☐ Yes<br>☐ No
2. Member of household	When was this person born?<br>*Please enter month and year only.*	Did this person have a corona test?
☐ Partner<br>☐ Daughter / son<br>☐ Mother / father<br>☐ Brother / sister<br>☐ Other person	Month: |__|__| Year: |__|__|__|__|	☐ Yes<br>☐ No
3. Member of household	When was this person born?<br>*Please enter month and year only.*	Did this person have a corona test?
☐ Partner<br>☐ Daughter / son<br>☐ Mother / father<br>☐ Brother / sister<br>☐ Other person	Month: |__|__| Year: |__|__|__|__|	☐ Yes<br>☐ No

For additional persons, use "template members of household“ at the end of this document.

(continue with question 21)

Achterstr. 30, 28359 Bremen Telefon 0800-247 72 45 Fax 0421 / 218-56821 E-Mail coverlauf@leibniz-bips.de

20. Do other people sleep in the same room as you?

- ☐ Yes *(continue with question 20a and 20b)*
- ☐ No *(continue with question 21)*

20a. How many people sleep in the same room as you, yourself not included?

Number |__|__|

20b. Did or do other persons who sleep in the same room as you have Corona (COVID-19) or did they have a positive Corona test?

- ☐ Yes
- ☐ No

21. Please enter the postal code of your residential address:

|__|__|__|__|__|

22. What is your highest degree of general education?
(only one answer is possible)

- ☐ I am still a student at a secondary school.
- ☐ I left school without a certificate.
- ☐ Basic secondary school certificate (German „Hauptschulabschluss/Volksschulabschluss“)
- ☐ Secondary school certificate (German „Realschulabschluss/Mittlere Reife“)
- ☐ Secondary school of the GDR with completed 8th or 9th grade (German „POS“)
- ☐ Secondary school of the GDR with completed 10th grade (German „POS“)
- ☐ A-levels for universities of applied sciences, completion of a technical secondary school (German „Fachhochschulreife/Abschluss einer Fachoberschule“)
- ☐ General or subject-related entrance qualification for higher education / A-levels (German „Abitur“, in the GDR „EOS/EOS mit Lehre“)
- ☐ Entrance qualification for higher education (German „Abitur“) after non-traditional schooling later on in life
- ☐ A different certificate *(continue with question 22a)*
- ☐ Don’t know
- ☐ No answer

(continue with question 23)

Achterstr. 30, 28359 Bremen Telefon 0800-247 72 45 Fax 0421 / 218-56821 E-Mail coverlauf@leibniz-bips.de

22a. You indicated that you received a different certificate. Please state the name of the certificate of general education.

____ *(free text)*

23. Which professional qualifications do you have?
If several answers apply, please indicate the highest vocational qualification.

- ☐ Still in vocational training [practical year, trainee, intern, student]
- ☐ Pupil and attending vocational secondary school, technical college, etc.
- ☐ No vocational qualification and not in vocational training
- ☐ Vocational training at a company [apprenticeship] completed
- ☐ Vocational schooling completed [vocational school, commercial school, employment in preparation of mid-level service in public administration]
- ☐ Training at a technical school in the GDR completed
- ☐ Completed training at a technical college, "master craftsman" training or technical school, vocational or technical academy
- ☐ Bachelor's degree from a (technical) university
- ☐ Degree from a university of applied sciences [e.g., diploma, master's degree]
- ☐ University degree [e.g., diploma, magister's degree, state board examination, master's degree]
- ☐ Dissertation
- ☐ Another professional qualification *(continue with question 23a)*
- ☐ Don't know
- ☐ No answer

(continue with question 24)

23a. You indicated that you have another professional qualification. Please state the name of the professional qualification.

____ *(free text)*

Achterstr. 30, 28359 Bremen Telefon 0800-247 72 45 Fax 0421 / 218-56821 E-Mail coverlauf@leibniz-bips.de

24. Are you currently working?

If several answers apply, please select the answer that fits the most.

- ☐ Full-time employment *(continue with question 25)*
- ☐ Part-time employment *(continue with question 25)*
- ☐ Partial retirement [regardless of whether currently in work or leave phase] *(continue with question 25)*
- ☐ Minor employment, “450-Euro-job” or so-called mini-job *(continue with question 25)*
- ☐ “One-Euro-job” [while receiving unemployment benefits (German „Arbeitslosengeld II“)] *(continue with question 25)*
- ☐ Occasionally or irregularly employed
- ☐ In vocational training/apprenticeship *(continue with question 25)*
- ☐ In retraining
- ☐ Federal voluntary service (German „Bundesfreiwilligendienst“), voluntary social/ecological year
- ☐ Maternity leave, childcare leave, parental leave or other leave
- ☐ Not employed [including: pupils or students not working for money, unemployed persons, early retirees, pensioners without supplementary income]
- ☐ Don’t know
- ☐ No answer

25. What is your current profession?

____ *(free text)*

26. If there are any comments you would like to share with us, please enter them in the following field. Please do not enter names, addresses or any other personal information to ensure data protection. Please be aware, that due to data protection rules we will be not able to contact you in order to provide answers to any comments or questions.
If you had more than one positive test result for SARS-CoV2 and these tests were more than 2 weeks apart from each other, please list the dates of all tests here. If you know the reason for these multiple tests, please add this information too. Please only list PCR-Tests (i.e., not rapid antigen tests or blood tests).

____

____

____

____*(free text)*

Thank you very much for your support! You have made an important contribution to research into the Corona disease (COVID-19).

Achterstr. 30, 28359 Bremen Telefon 0800-247 72 45 Fax 0421 / 218-56821 E-Mail coverlauf@leibniz-bips.de

CoVerlauf

Leibniz-Institut für Präventionsforschung und Epidemiologie – BIPS GmbH

„Template: Members of household“

Addition to question 19c for households with more than 3 members

4. Member of household	When was this person born?<br>*Please enter month and year only.*	Did this person have a corona test?
☐ Partner<br>☐ Daughter / son<br>☐ Mother / father<br>☐ Brother / sister<br>☐ Other person	Month:|__|__| Year: |__|__|__|__|	☐ Yes<br>☐ No
5. Member of household	When was this person born?<br>*Please enter month and year only.*	Did this person have a corona test?
☐ Partner<br>☐ Daughter / son<br>☐ Mother / father<br>☐ Brother / sister<br>☐ Other person	Month:|__|__| Year: |__|__|__|__|	☐ Yes<br>☐ No
6. Member of household	When was this person born?<br>*Please enter month and year only.*	Did this person have a corona test?
☐ Partner<br>☐ Daughter / son<br>☐ Mother / father<br>☐ Brother / sister<br>☐ Other person	Month:|__|__| Year: |__|__|__|__|	☐ Yes<br>☐ No
7. Member of household	When was this person born?<br>*Please enter month and year only.*	Did this person have a corona test?
☐ Partner<br>☐ Daughter / son<br>☐ Mother / father<br>☐ Brother / sister<br>☐ Other person	Month:|__|__| Year: |__|__|__|__|	☐ Yes<br>☐ No
8. Member of household	When was this person born?<br>*Please enter month and year only.*	Did this person have a corona test?
☐ Partner<br>☐ Daughter / son<br>☐ Mother / father<br>☐ Brother / sister<br>☐ Other person	Month:|__|__| Year: |__|__|__|__|	☐ Yes<br>☐ No

Achterstr. 30, 28359 Bremen Telefon 0800-247 72 45 Fax 0421 / 218-56821 E-Mail coverlauf@leibniz-bips.de

9. Member of household	When was this person born? *Please enter month and year only.*	Did this person have a corona test?
☐ Partner<br>☐ Daughter / son<br>☐ Mother / father<br>☐ Brother / sister<br>☐ Other person	Month:|__|__| Year: |__|__|__|__|	☐ Yes<br>☐ No
10. Member of household	When was this person born? *Please enter month and year only.*	Did this person have a corona test?
☐ Partner<br>☐ Daughter / son<br>☐ Mother / father<br>☐ Brother / sister<br>☐ Other person	Month:|__|__| Year: |__|__|__|__|	☐ Yes<br>☐ No
11. Member of household	When was this person born? *Please enter month and year only.*	Did this person have a corona test?
☐ Partner<br>☐ Daughter / son<br>☐ Mother / father<br>☐ Brother / sister<br>☐ Other person	Month:|__|__| Year: |__|__|__|__|	☐ Yes<br>☐ No
12. Member of household	When was this person born? *Please enter month and year only.*	Did this person have a corona test?
☐ Partner<br>☐ Daughter / son<br>☐ Mother / father<br>☐ Brother / sister<br>☐ Other person	Month:|__|__| Year: |__|__|__|__|	☐ Yes<br>☐ No

Achterstr. 30, 28359 Bremen Telefon 0800-247 72 45 Fax 0421 / 218-56821 E-Mail coverlauf@leibniz-bips.de
